# Heterologous co-expression of a yeast diacylglycerol acyltransferase (*ScDGA1*) and a plant oleosin (*AtOLEO3*) as an efficient tool for enhancing triacylglycerol accumulation in the marine diatom *Phaeodactylum tricornutum*

**DOI:** 10.1186/s13068-017-0874-1

**Published:** 2017-07-17

**Authors:** Nodumo Nokulunga Zulu, Jennifer Popko, Krzysztof Zienkiewicz, Pablo Tarazona, Cornelia Herrfurth, Ivo Feussner

**Affiliations:** 1 Department of Plant Biochemistry, Albrecht-von-Haller-Institute for Plant Sciences, University of Goettingen, 37077 Goettingen, Germany; 2 Novagreen Projektmanagement GmbH, 49377 Vechta, Germany; 3 Department of Plant Biochemistry, Goettingen Center for Molecular Biosciences (GZMB), University of Goettingen, 37077 Goettingen, Germany; 4 Department of Plant Biochemistry, International Center for Advanced Studies of Energy Conversion (ICASEC), University of Goettingen, 37077 Goettingen, Germany

**Keywords:** Biofuel, DGAT, Diatom, Lipid droplets, Microalgae, Neutral lipid biosynthesis

## Abstract

**Background:**

Microalgae are promising alternate and renewable sources for producing valuable products such as biofuel and essential fatty acids. Although this is the case, there are still challenges impeding on the effective commercial production of microalgal products. For instance, their product yield is still too low. Therefore, this study was oriented towards enhancing triacylglycerol (TAG) accumulation in the diatom *Phaeodactylum tricornutum* (strain Pt4). To achieve this, a type 2 acyl-CoA:diacylglycerol acyltransferase from yeast (*ScDGA1*) and the lipid droplet (LD) stabilizing oleosin protein 3 from *Arabidopsis thaliana* (*AtOLEO3*) were expressed in Pt4.

**Results:**

The individual expression of *ScDGA1* and *AtOLEO3* in Pt4 resulted in a 2.3- and 1.4-fold increase in TAG levels, respectively, in comparison to the wild type. The co-expression of both, *ScDGA1* and *AtOLEO3*, was accompanied by a 3.6-fold increase in TAG content. On the cellular level, the lines co-expressing *ScDGA1* and *AtOLEO3* showed the presence of the larger and increased numbers of lipid droplets when compared to transformants expressing single genes and an empty vector. Under nitrogen stress, TAG productivity was further increased twofold in comparison to nitrogen-replete conditions. While TAG accumulation was enhanced in the analyzed transformants, the fatty acid composition remained unchanged neither in the total lipid nor in the TAG profile.

**Conclusions:**

The co-expression of two genes was shown to be a more effective strategy for enhancing TAG accumulation in *P. tricornutum* strain Pt4 than a single gene strategy. For the first time in a diatom, a LD protein from a vascular plant, oleosin, was shown to have an impact on TAG accumulation and on LD organization.

**Electronic supplementary material:**

The online version of this article (doi:10.1186/s13068-017-0874-1) contains supplementary material, which is available to authorized users.

## Background

In the past years, microalgal products have received a great deal of attention because of their potential in resolving various problems encountered in environmental and health sectors [1, 2]. The fast growth rates and the ability to accumulate high yields of lipids have made microalgae better alternate sources for biofuel production in comparison to plants [3–6]. Furthermore, microalgae are capable of producing very high amounts of essential very long-chain polyunsaturated fatty acids (VLC-PUFAs) such as arachidonic acid (ARA), eicosapentaenoic acid (EPA), and docosahexaenoic acid (DHA) [3]. Despite the fact that microalgae have several advantages over plants as sources of biofuel and other highly valuable products, the microalgal industry is still not economically feasible [7]. One of the major bottlenecks of this industry is the trade-off between biomass production and lipid productivity as the strategies that are commonly used to enhance lipid accumulation impede biomass production [8]. Furthermore, product recovery from microalgal biomass is usually an expensive process [7]. Therefore, it is often preferred that microalgal products such as the essential VLC-PUFAs are deposited in triacylglycerols (TAGs), which are a preferred form of storage in terms of downstream processing [9].

TAGs can be synthesized via two different pathways, the acyl-CoA dependent de novo synthesis (also known as part of the Kennedy pathway) and the acyl-CoA independent pathway by degrading primarily existing organelle membranes [8]. In the Kennedy pathway, the last step of TAG synthesis involves the acylation of diacylglycerol (DAG) by an acyl-CoA:diacylglycerol acyltransferase (DGAT), whereas in the latter pathway DAG is acylated by the phospholipid:diacylglycerol acyltransferase (PDAT). Among DAGTs, the two mostly studied groups of these enzymes belong to type 1 and type 2. Although both types of DGATs perform similar functions, their sequences and preferences are different due to separate evolution [10]. In plants, both types of DGATs exist, and DGAT1 has been shown to be a major contributor of TAG accumulation in seeds [11, 12]. In contrast, the baker’s yeast (*Saccharomyces cerevisiae*) only has a type 2 DGAT that is commonly known as *ScDGA1* [13]. *ScDGA1* has been previously shown to have a broad substrate specificity, in terms of the fatty acids it incorporates into TAGs [14]. This feature makes it a DGAT of interest in cases where the substrate pool is not well defined. Like plants and animals, microalgae have both types of DGATs. Remarkably, some microalgal species have multiple copies of DGAT2 genes [15]. For instance, *P. tricornutum* has 4 copies of DGAT2, whereas *Nannochloropsis oceanica* has 13 copies of DGATs, of which 12 encode DGAT2 [16]. Furthermore, DGAT2 from different species of microalgae exhibit diverse substrate specificities, which is reflected in the fatty acid composition of TAG among algal genera. Some microalgal species have high levels of VLC-PUFAs in their TAGs while others only have high amounts of unsaturated 16 carbon fatty acids. For example, *Ostreococcus tauri* accumulates high levels of DHA in TAGs [4, 14], whereas in *P. tricornutum* this VLC-PUFA is almost excluded from the TAGs [17].

Following their synthesis, TAGs are deposited in lipid droplets (LDs), which consist of a hydrophobic core that is surrounded by a monolayer of phospholipids with few embedded specific proteins [18]. LDs have a wide range of surface proteins, and in plants, oleosins have been found to be the most abundant proteins associated with the LDs in seeds [19]. Oleosins have been shown to stabilize LDs and prevent them from coalescing and degradation by providing steric hindrance and minimizing the access of TAG lipases, respectively, in seeds of flowering plants [18, 19]. Although oleosins are not present in microalgae, various other LD associated proteins have been characterized from various microalgal species [20, 21]. These include a major lipid droplet protein (MLDP) from *Chlamydomonas reinhardtii* [22], a lipid droplet surface protein (LDSP) from *Nannochlorospsis* sp. [23] and a stramenopile-type lipid droplet protein (StLDP) from *P.* *tricornutum* [20].

In this study, the diatom *P. tricornutum* was used as a model for engineering lipid metabolism. Diatoms are receiving a great deal of attention as a platform to produce biofuel [24], because they store TAGs as other microalgae as the major carbon source upon stress, i.e., when nitrogen becomes limiting. However, diatoms are better suited for biofuel production when compared to other classes of microalgae such as the chlorophytes because their fatty acid profiles are enriched with up to 80% of medium and long chain saturated and monounsaturated fatty acids of 14–16 carbon atoms [25]. In contrast, chlorophytes mostly have high levels of polyunsaturated long chain fatty acids, mostly C18 fatty acids. For biofuel production, medium chain saturated fatty acids are preferred because they increase the ignition quality of biofuels [25]. In addition to being good sources of biofuel, diatoms are also a source of the essential *ω*3 fatty acid EPA, which is not prevalent in chlorophytes [25]. In addition, they also produce pigments such as carotenoids, which have applications in the pharmaceutical, food, and cosmeceutical industries [26, 27].

*Phaeodactylum tricornutum* is one of the mostly studied diatoms, and in this study strain Pt4 was used as a model. Pt4 was isolated at the Island of Segelskår, Finland, which has a lower salt concentration in comparison to the regions of isolation for the other strains of *P. tricornutum* [28]. Therefore, Pt4 can be cultivated in industrial production systems close to biogas fermentation plants that are located far from coastal areas where salt water is not immediately available [17]. Although Pt4, accumulates low levels of TAGs in comparison to the other *P. tricornutum* strains [28], this opens up a platform for establishing higher TAG contents in a marine strain that can be cultivated at industrial scale without the risk of equipment corrosion caused by high salt content. In order to enhance TAG accumulation in Pt4, we have expressed a type 2 DGAT from *S. cerevisiae* (*ScDGA1*). We also pursued the possibility of further increasing TAG productivity by blocking TAG degradation through the co-expression of *ScDGA1* and oleosin 3 from *A.* *thaliana* (*AtOLEO3*). We first assessed the individual expression of these genes in terms of TAG accumulation and then we compared this effect to data obtained when both, *ScDGA1* and *AtOLEO3*, are co-expressed in Pt4. Our results demonstrated that the co-expression of *ScDGA1* and *AtOLEO3* is a more effective approach towards enhancing TAG accumulation in Pt4 than the expression of single genes and demonstrates the potential of heterologous gene expression in microalgae as a tool for enhancing the TAG content.

## Results

### The expression of *ScDGA1* results in higher lipid accumulation

**Fig. 1:**
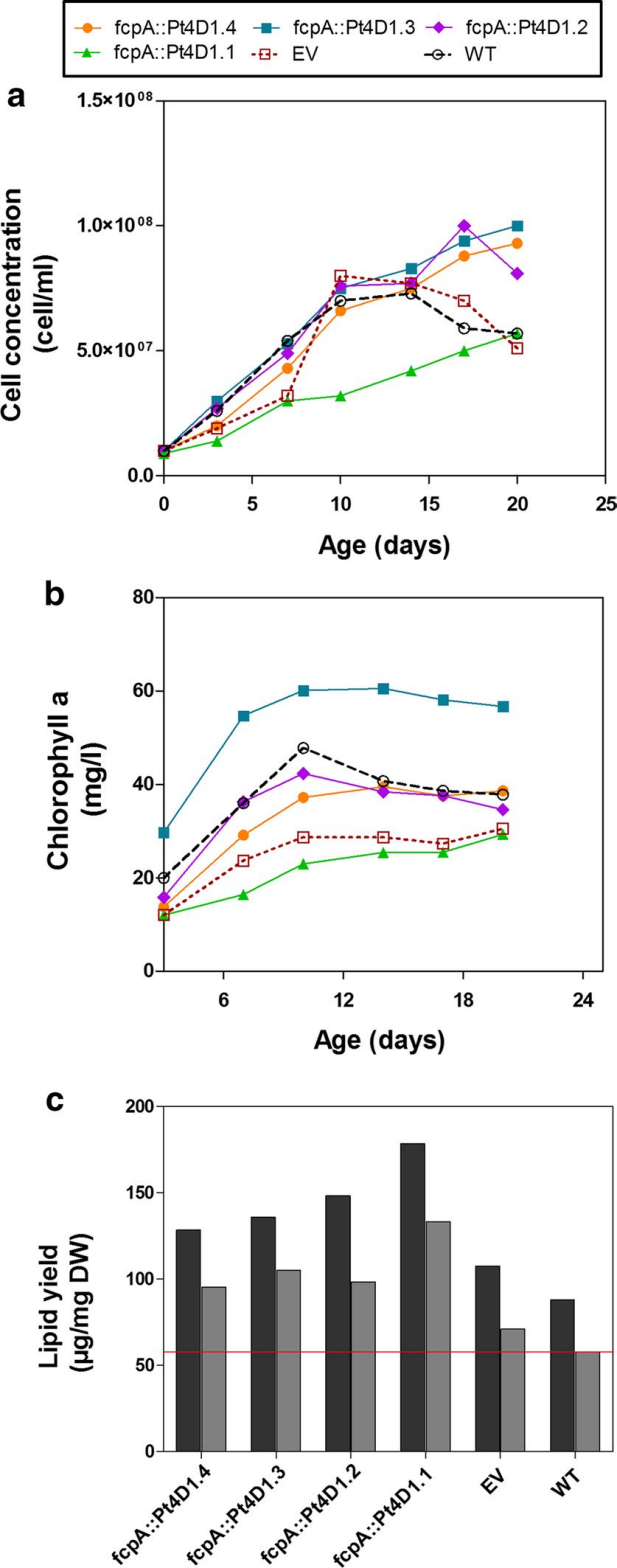
Assessing the effect of expressing *ScDGA1* in Pt4 on growth and lipid accumulation. Growth profiles for these lines are based on cell concentration (**a**) and chlorophyll a content (**b**). Data points on the profiles represent mean values from two independent studies, where in each study there were two biological replicates. Lipid yield in the lines is shown as maximum lipid yield obtained from total lipids and TAGs (**c**) from day 7 of culture when transformants showed the highest TAG levels. *EV* empty vector, *TAG* triacylglycerol, *WT* wild type. Raw data for growth profiles and lipid yields can be obtained from Additional file 14

*ScDGA1* has been previously demonstrated to have a broad substrate specificity, in terms of fatty acids it can incorporate into TAGs [14, 29]. For that reason, this type 2 DGAT was expressed in Pt4 towards achieving the aim of enhancing TAG accumulation in the background of an unknown substrate pool. The expression of *ScDGA1* in Pt4 was driven by the native fucoxanthin chlorophyll binding protein A (fcpA) promoter. This endogenous promoter is frequently used to drive stable gene expression in *P. tricornutum* and it is active during the photosynthetic phase of growth [30]. The resulting transformants were subjected to semi-quantitative PCR for confirming the expression of *ScDGA1* (Additional file 1: Figure S1). Of the eight lines that showed positive *ScDGA1* gene expression, four were randomly selected for further characterization. The assessment of growth profiles showed that there were no differences in growth patterns between the lines, empty vector control line and wild type. However, with the exception of line Pt4D1.1, which showed a lower cell concentration and chlorophyll a content (Fig. 1a, b). Regardless of its lower cell concentration, Pt4D1.1 accumulated the highest yields of total lipids and TAGs, reaching levels of 178 and 133 µg/mg, respectively, in comparison to the other lines and controls on day 7 (Fig. 1c). This corresponded to a twofold increase in total lipids and in TAGs in comparison to the wild type. For this reason, this line (Pt4D1.1) was selected for further analysis. It was also noted that in all Pt4 lines expressing the *ScDGA1* gene, there were no changes in fatty acid composition in total lipids or TAGs (Additional file 2: Figure S2).

### The expression of *AtOLEO3* does not affect the growth of Pt4 but has a positive impact on TAG accumulation

**Fig. 2:**
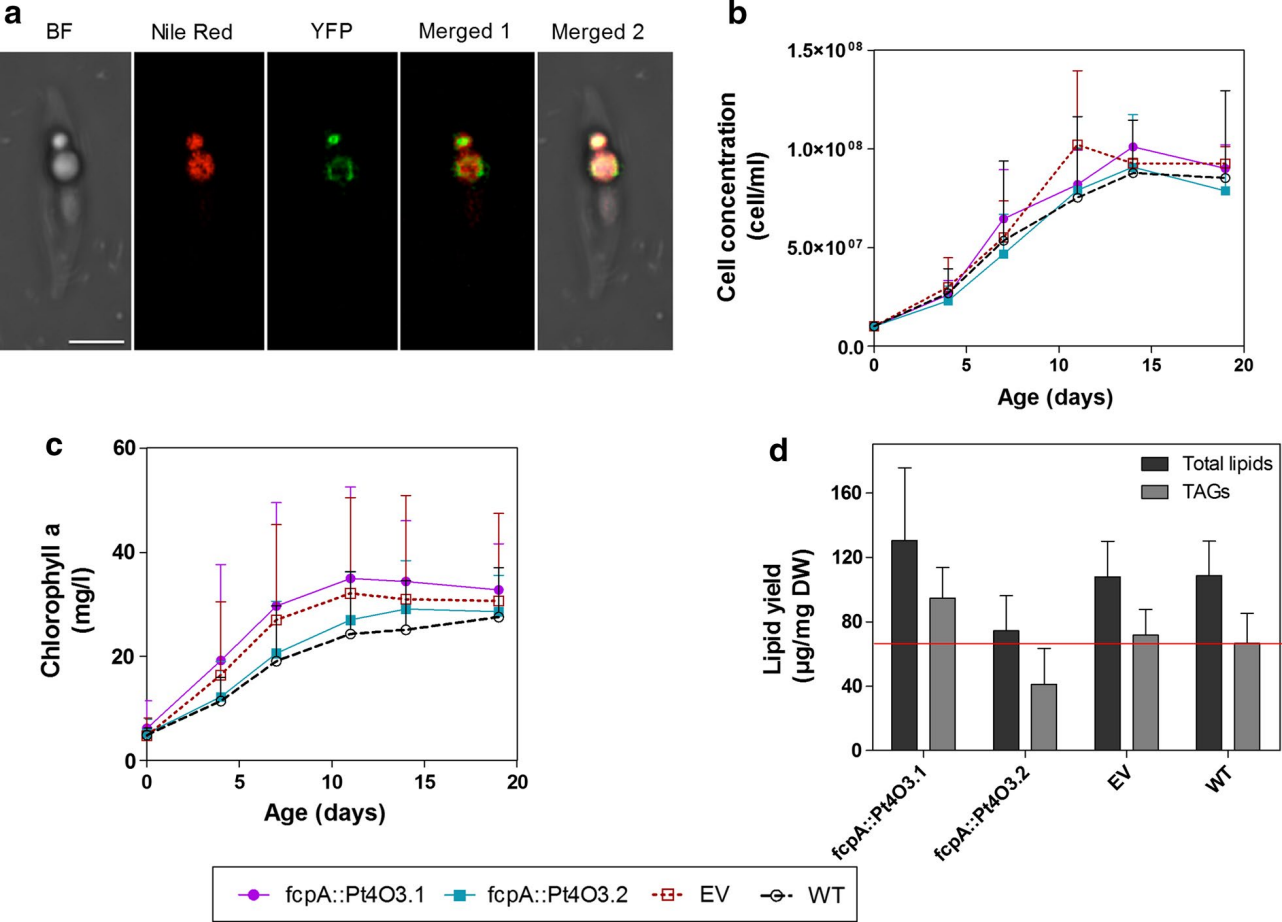
The impact of AtOLEO3 on growth and lipid accumulation in Pt4. **a** Localization of AtOLEO3-YFP construct into LDs in Pt4. Growth profiles for the transgenic lines based on cell concentration (**b**) and chlorophyll a content (**c**). The data points on these profiles represent mean values from three independent studies, where in each study there were two biological replicates. **d** The impact of ScDGA1 on lipid yield is shown as maximum lipid yield obtained from total lipids and TAGs. Yields were obtained from day 14 of culture; where transformants showed the highest TAG levels. *EV* empty vector, *TAG* triacylglycerol, *WT* wild type. *Scale bar* 5 µm. Raw data for growth profiles and lipid yields can be obtained from Additional file 14

Oleosins have been shown to specifically localize at the seed LDs of plants and are thought to stabilize and protect LDs from degradation [31]. Thus, since there are no oleosins in microalgae, we expressed the oleosin 3 gene from *A. thaliana* in order to test if its function to stabilize LDs is also conserved in other systems. To verify the localization of *AtOLEO3* in Pt4, the *AtOLEO3* encoding gene was tagged to YFP. The microscopic analysis showed that *AtOLEO3* specifically localized to the LDs when expressed in Pt4; this is indicated by the YFP signal surrounding the LDs (Fig. 2a). In a separate study, the randomly selected lines expressing non-tagged *AtOLEO3* (Pt4O3.1 and Pt4O3.2) were analyzed for growth rates and TAG accumulation. *AtOLEO3* gene expression was confirmed in these lines (Additional file 3: Figure S3). The obtained results showed that the lines grew in the same manner as the empty vector and wild type (Fig. 2b, c) but in terms of lipid accumulation, it was observed that line Pt4O3.1 accumulated more TAGs and total lipids, when compared to the empty vector and wild type. It reached 94.5 and 130.4 µg/mg, TAGs and total lipids, respectively, in comparison to the other line and controls at day 14 (Fig. 2d). This corresponded to a 1.4- and 1.2-fold increase in TAGs and total lipids respectively. Surprisingly, line Pt4O3.2 of the *AtOLEO3* transformants accumulated even less total lipids and TAGs when compared to the empty vector control and wild type. This could have been due to random integration of *AtOLEO3* at a critical site in the genome of Pt4. It was also noted that in both lines, the expression of *AtOLEO3* in Pt4 did not have any influence on the fatty acid composition neither of TAGs nor of total lipids (Additional file 4: Figure S4).

### Co-expression of *ScDGA1* and *AtOLEO3* further increases TAG accumulation

**Fig. 3:**
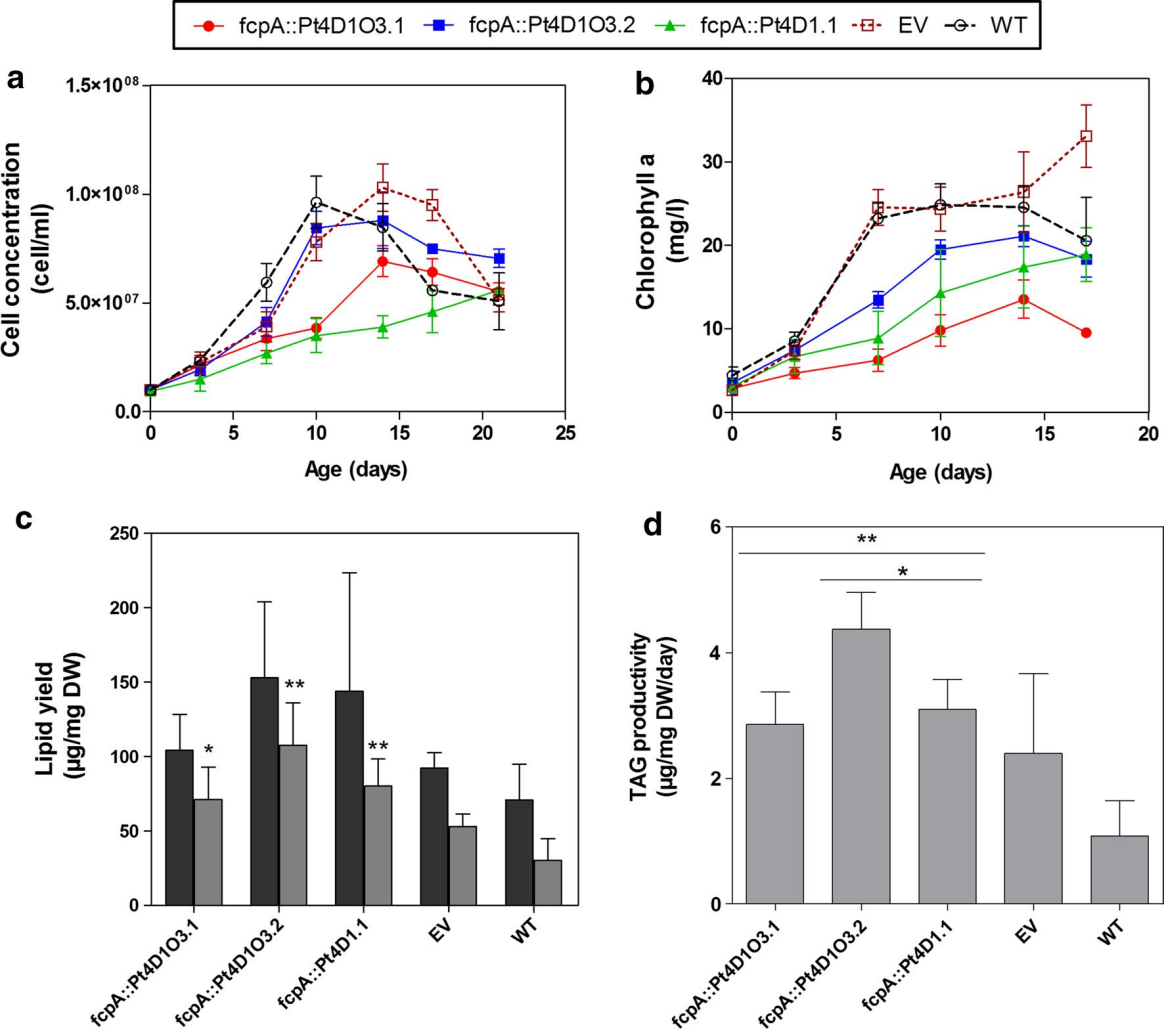
The impact of co-expressing ScDGA1 and AtOLEO3 in Pt4 on growth and lipid accumulation in Pt4. Growth profiles for the transformants are based on cell concentration (**a**) and chlorophyll a content (**b**). The data points on these profiles represent a mean value from three independent studies, where in each study there were two biological replicates. Maximum lipid yield obtained from total lipids (*black boxes*) and TAGs (*gray boxes*) was also assessed for multigene and single gene expressing transformants (**c**). These yields were obtained from day 17; where transformants showed the highest yields. TAG productivities were calculated from maximum TAG yields (**d**). The values for these bar graphs represent mean values from three independent studies; in each study there were two biological replicates. Error bars were calculated from the standard deviation. *p* values were calculated from the student’s *t* test, where (*asterisk*) means *p* ≤ 0.05 and (*double asterisk*) means *p* ≤ 0.01. *EV* empty vector, *TAG* triacylglycerol, *WT* wild type. Raw data for growth profiles, lipid yields and TAG productivities can be obtained from Additional file 14

The co-expression of a DGAT and a stabilizing LD protein had been shown to have a positive impact on TAG accumulation in the leaves of *A. thaliana* [32]. To our knowledge, the co-expression of DGAT and oleosin has not yet been demonstrated in microalgae. Thus, we tested the impact of co-expressing *ScDGA1* and *AtOLEO3* on growth rates and TAG levels in Pt4 under the control of the fcpA promoter. We selected two lines, (Pt4D1O3.1 and Pt4D1O3.2) showing positive gene expression for *ScDGA1* and *AtOLEO3* (Additional file 5: Figure S5), for analysis in terms of growth and lipid accumulation. The performance of these co-expressing lines was then assessed in parallel to line Pt4D1.1 (only expressing *ScDGA1*), in terms of growth rates and lipid accumulation (Fig. 3a, b). Similar to line Pt4D1.1, both co-expressing lines showed lower growth rates when compared to the empty vector line and the wild type (Fig. 3a, b). The highest total lipid and TAG yields were obtained from line Pt4D1O3.2, reaching 152 and 107 µg/mg, respectively (Fig. 3c); thus corresponding to a 2.2- and 3.6-fold increase in total lipids and TAGs, respectively. The differences in TAG yields, especially between the double and single gene expressing lines, were also observed at the level of TAG productivities (Fig. 3d), where the best strategy would produce the highest TAG levels in a short period of time. As shown in Fig. 3d, all three transgenic lines displayed significantly higher levels of TAG productivities in comparison to the wild type; TAG productivities were increased by 2.2-, 3.5-, and 2.5-folds for Pt4D1O3.1, Pt4D1O3.2, and Pt4D1.1, respectively. Moreover, it was observed that line Pt4D1O3.2 (co-expressing *ScDGA1* and *AtOLEO3*) had a 1.4-fold higher TAG productivity (4.38 µg/mg/day) when compared to line Pt4D1.1 (3.1 µg/mg/day), which only expressed *ScDGA1* (Fig. 3d). Overall, these results strongly suggest that the co-expression of *ScDGA1* and *AtOLEO3* results in a further enhancement of TAG accumulation in Pt4, in comparison to when *ScDGA1* is expressed on its own.

**Fig. 4:**
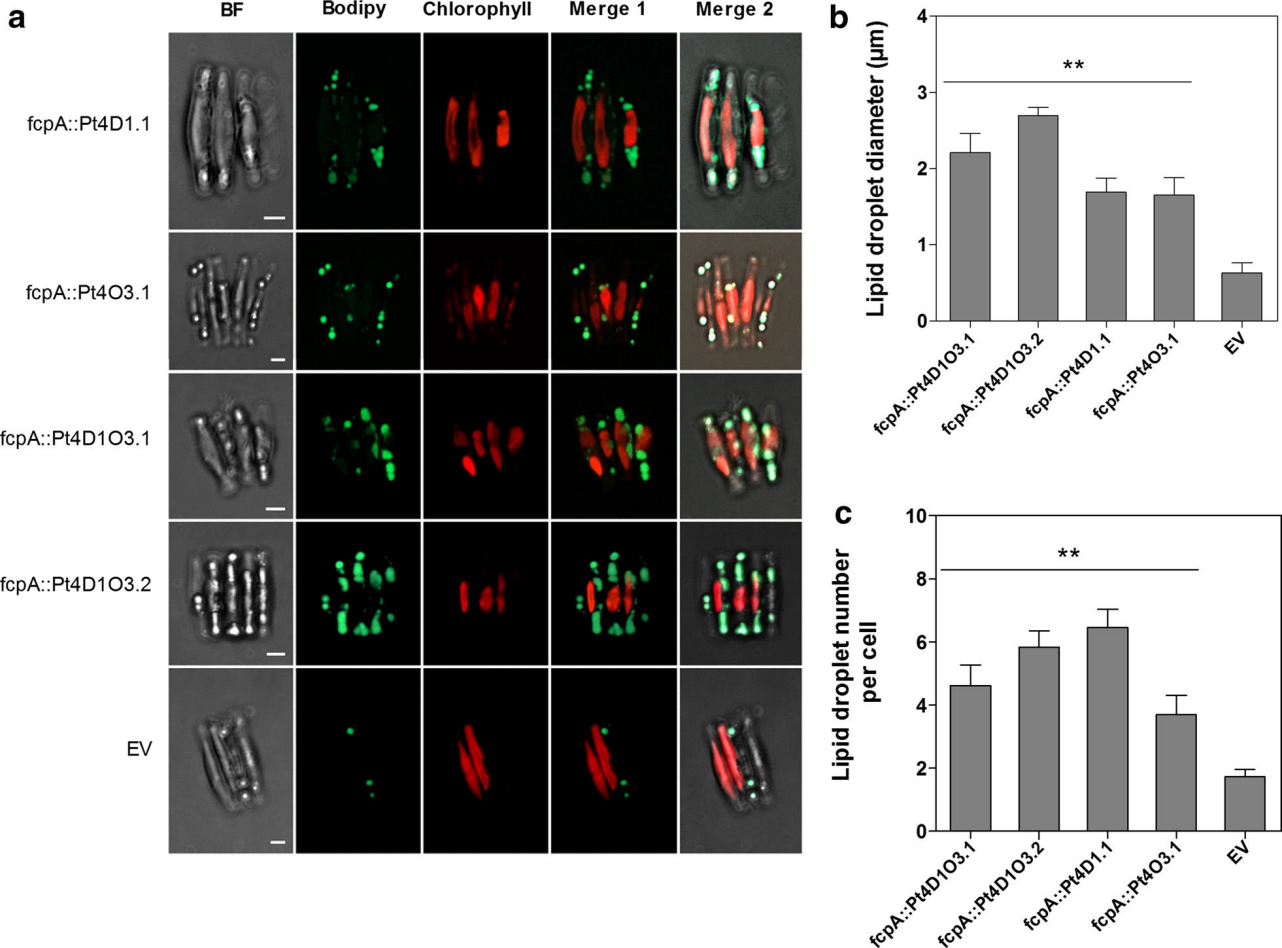
Assessing the effect of expressing AtOLEO3 in Pt4 on LD number and diameter. The LD analysis was done for the single gene expressing lines, Pt4D1.1 and line Pt4O3.1 as well as for the double gene co-expressing lines Pt4D1O3.1 and Pt4D1O3.2; the EV line was used as a control (**a**). These were analyzed after BODIPY 493/504 staining of cells at day 14 in the growth phase. Images are composites of BF, BODIPY 493/504 fluorescence (*green*), chlorophyll autofluorescence (*red*) and merged images. *Scale bar* 5 µm. The mean values on the bar graphs represent LD diameter (**b**) and number (**c**). The mean values are a representative of three technical replicates and the error bars were calculated from the standard deviation. *p* values were calculated from the student’s *t* test, where (*double asterisk*) means *p* ≤ 0.01. *BF* bright field, *EV* empty vector, *LD* lipid droplet. Raw data for LD diameter and number per cell can be obtained from Additional file 14

In order to have a better understanding of the impact of *ScDGA1* and *AtOLEO3* genes on the formation of LDs, we analyzed the behavior of LDs in Pt4 lines co-expressing *ScDGA1* and *AtOLEO*3 (lines Pt4D1O3.1 and Pt4D1O3.2) as well as lines individually expressing *ScDGA1* (Pt4D1.1) and *AtOLEO3* (Pt4O3.1), at cellular level (Fig. 4). This analysis showed that in comparison to the empty vector control, the individual expression of *ScDGA1* (Pt4D1.1) and *AtOLEO*3 (Pt4O3.1) in Pt4 resulted in the formation of an increased number of LDs with an average diameter of 1.69 and 1.65 µm, respectively (Fig. 4a, b). In contrast, the co-expression of *ScDGA1* and *AtOLEO3* (Fig. 4a, for lines Pt4D1O3.1 and Pt4D1O3.2) was accompanied by the formation of prominent LDs, often of irregular shape. In these lines, the LDs occupied most of the cell volume and their average diameter reached 2.21 and 2.56 µm in lines Pt4D1O3.1 and Pt4D1O3.2, respectively (Fig. 4b). These large LDs in the lines co-expressing *ScDGA1* and *AtOLEO3* were however less numerous, when compared to single gene expressing lines (Fig. 4c).

Despite the significant increase in TAG yields, there were no changes observed in the fatty acid composition in response to the co-expression of *ScDGA1* and *AtOLEO3* genes (Additional file 6: Figure S6). Similarly, there were no changes observed in the distribution of TAG molecular species (Additional file 7: Figure S7). TAG distribution remained similar between the transgenic lines and empty vector control. TAG species containing the saturated and monounsaturated fatty acids were still the major contributors in the TAG pool in all three lines (Pt4D1O3.1, Pt4D1O3.2, and Pt4D1.1). In addition, there were no new TAG species introduced by *ScDGA1*. Through the summing up of all detected TAG molecular species, we could validate our GC data, which showed that line Pt4D1O3.2 had accumulated more TAGs than the other lines. In addition to analyzing TAG molecular species, the acyl-CoA pool was also assessed to check for any possible substrate limitations. This analysis was only carried out for the highest TAG accumulating line co-expressing *ScDGA1* and *AtOLEO3* (Pt4D1O3.2) and compared to the empty vector and wild type. Again, we found no significant differences in the acyl-CoA pool (Additional file 8: Figure S8).

### Nitrogen starvation induces additional increase of TAG content in Pt4 co-expressing *ScDGA1* and *AtOLEO3*

**Fig. 5:**
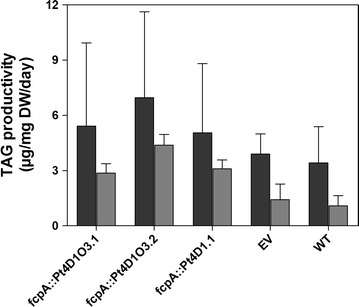
Effect of nitrogen starvation on TAG productivity in transgenic lines of Pt4. This profile shows maximum TAG productivities under nitrogen replete and deplete conditions. Data points on these profiles represent the mean values from three independent studies where in each independent study there were two biological replicates. Error bars were calculated from the standard deviation. *EV* empty vector, *TAG* triacylglycerol, *WT* wild type. Raw data for TAG productivities can be obtained from Additional file 14

Nitrogen (N) stress has been widely reported to influence TAG accumulation in microalgae [33]. Therefore, there was an interest in determining if TAG accumulation could be further enhanced in the obtained Pt4 lines, in response to N starvation. When exposed to N stress, lines Pt4D1O3.1 and Pt4D1O3.2 as well as Pt4D1.1 showed a further increment in TAG levels (Fig. 5). Interestingly, a similar pattern of TAG productivities was observed under both, N deplete and N replete conditions with line Pt4D1O3.2 showing the highest TAG productivity (6.9 µg/mg/day) in comparison to lines Pt4D1O3.1 (5.41 µg/mg/day) and Pt4D1.1 (5.06 µg/mg/day) (Fig. 5). Although it is clear that N stress further enhanced TAG productivities in the analyzed lines of Pt4, statistical analysis could not be performed due to the fact that the deviation between the samples exposed to N stress was high, possibly a consequence of increased sedimentation of Pt4 cells under these conditions.

### Assessing the impact of the NR promoter in driving gene expression of *ScDGA1* and *AtOLEO3*

**Fig. 6:**
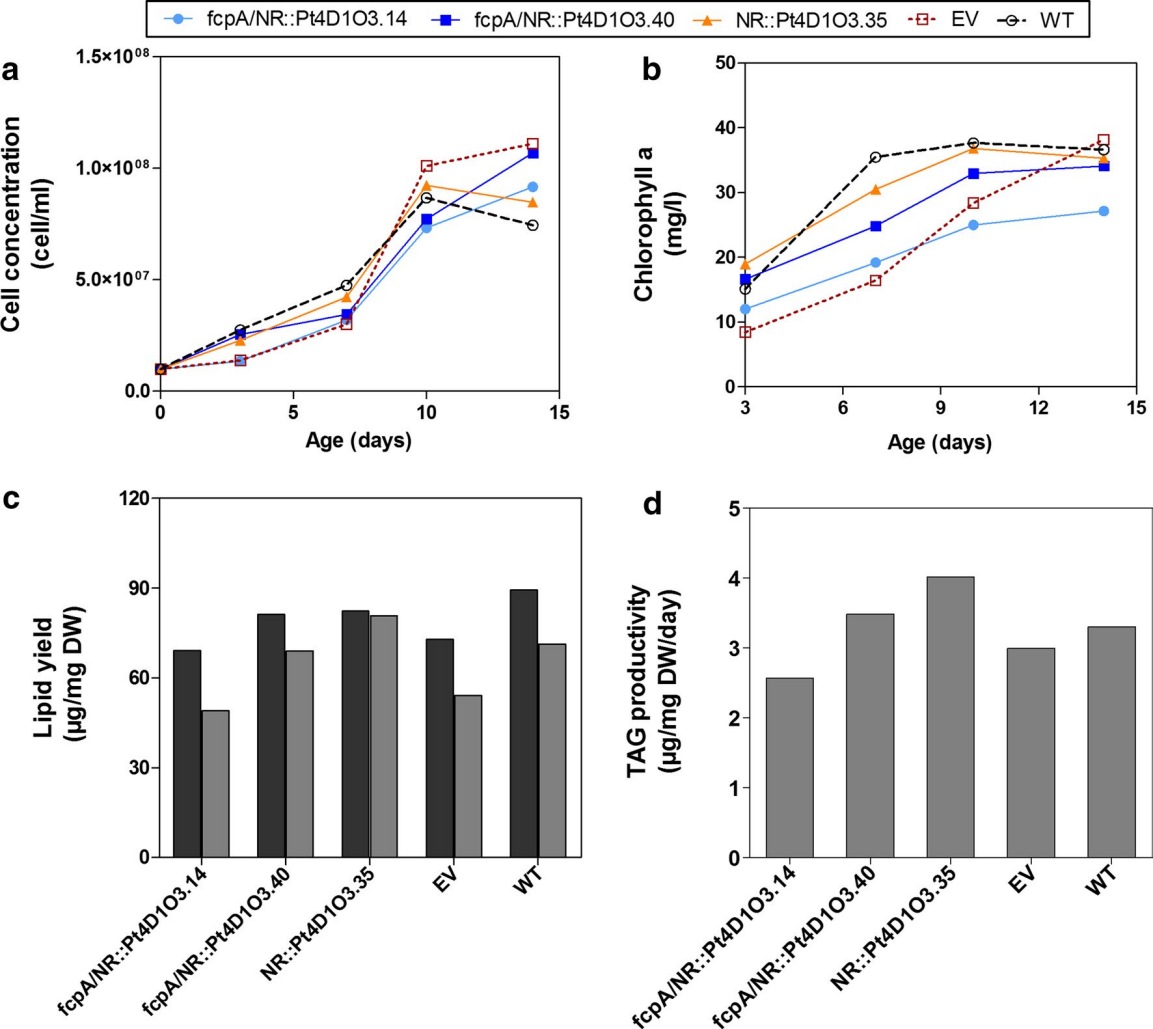
The impact of the NR promoter on growth and lipid yield in lines co-expressing *ScDGA1* and *AtOLEO3*. Growth profiles are based on cell concentration (**a**) and chlorophyll a content (**b**). Maximum lipid yields (**c**) were obtained from day 17, a time point where the highest TAG yields were accumulated and TAG productivities (**d**) were calculated from maximum TAG yields. The bar graphs represent mean values of two independent studies, where in each study there were two biological replicates. *EV* empty vector, *NR* nitrate reductase, *TAG* triacylglycerol, *WT* wild type. Raw data for growth profiles, lipid yields, and TAG productivities can be obtained from Additional file 14

The nitrate reductase (NR) promoter is yet another endogenous promoter frequently used to drive gene expression in *P.* *tricornutum* [34]. Although this promoter has recently been reported to be stronger than the fcpA promoter at driving stable gene expression in *P. tricornutum* [34], when we expressed YFP under both promoters (fcpA and NR) in Pt4, there appeared to be no differences in the YFP signal intensity (Additional file 9: Figure S9). Nonetheless, we also generated transformants expressing *ScDGA1* and *AOLEO3* under the NR promoter, in order to determine if there would be differences in TAG accumulation when compared to the lines expressing *ScDGA1* and *AOLEO3* genes under the control of the fcpA promoter. From the randomly selected lines, three of them tested positive for gene expression (Additional file 10: Figure S10). Lines Pt4D1O3.14 and Pt4D1O3.40 were expressing *ScDGA1* under both promoters (fcpA and NR), such that there were two copies of *ScDGA1* in one construct and *AtOLEO3* was only expressed under the NR promoter. Line Pt4D1O3.35 was co-expressing *ScDGA1* and *AtOLEO3* under the NR promoter. There were no significant differences in growth behavior between the transgenic lines and the controls (Fig. 6a, b). The analysis of lipid yields showed that line Pt4D1O3.35 (*ScDGA1* and *AtOLEO3* under NR promoter) accumulated more TAGs than the other two lines and the controls (Fig. 6c). Consequently, this effect was also visible when TAG productivities were compared since the line Pt4D1O3.35 had the highest TAG productivity of all analyzed lines (Fig. 6d). Noteworthy, the TAG productivity of line Pt4D1O3.35 obtained under the NR promoter (4.01 µg/mg/day) was only slightly lower than TAG productivity obtained under the fcpA promoter (4.4 µg/mg/day, as previously shown in Fig. 3d).

## Discussion

The aim of this study was to improve TAG accumulation in *P. tricornutum*, strain Pt4, which naturally grows in less saline conditions in comparison to the other *P. tricornutum* strains [17, 28]. To achieve this aim, a gene encoding an enzyme catalyzing the last and only specific metabolic step in TAG formation, DGAT2 from the baker’s yeast (*ScDGA1*), together with a gene encoding the LD stabilizing protein oleosin 3 from *A. thaliana* (*AtOLEO3*) were expressed in Pt4, respectively. Oleosins in general have been shown to maximize TAG accumulation in plants, most likely by preventing the exposure of TAGs to TAG lipases [31]. *AtOLEO3* was selected because of its hampering effect on TAG degradation [32]. *ScDGA1* was selected to be expressed in Pt4 because of its broad substrate specificity [13, 14, 29, 35] and when expressed individually in Pt4, *ScDGA1* and *AtOLEO3* resulted in different responses in terms of growth rates and lipid accumulation. Individual expression of *ScDGA1* (line Pt4D1.1) accumulated the highest amounts of TAGs in comparison to the other *ScDGA1* lines and controls (Fig. 1c), reaching a 50% increase in TAG levels, when compared to the empty vector control. This promising increase in TAG accumulation was however accompanied by an impaired growth rate (Fig. 1a, b). Since this was the only line out of four independent events that showed this characteristics it is not clear if this was a result of *ScDGA1* expression or just a possible artifact caused by random integration of *ScDGA1* at a critical site in the genome of Pt4. However, it was previously observed that improving TAG synthesis in microalgae results in reduced growth rate [36], as the cells store more carbon instead of using it for growth. This was also the case when glycerol-3-phosphate dehydrogenase (G3PDH) was overexpressed in *P. tricornutum*, where this overexpression improved TAG accumulation but slowed down the growth rate [1]. Although the physiological nature of this conundrum still remains unknown, it seems likely that the changes in cellular carbon flux could potentially trigger yet unknown cellular energy stress-related pathways resulting in an impaired cell cycle and cell division. Contrary to this, the overexpression of the native DGAT2 in *P.* *tricornutum* was reported to increase neutral lipid levels without affecting biomass production [37]. Thus, it is possible that the observed negative correlation between oil accumulation and growth rates in Pt4 could simply be a result of heterologous gene expression, which affects the metabolism and developmental program of the host cell. Interestingly, the expression of *AtOLEO3* in Pt4 (Pt4O3.1) resulted in an increased TAG accumulation and no differences in growth patterns were observed in comparison to the wild type and empty vector (Fig. 2b, c). Furthermore, total lipids and TAG levels were enhanced in this line in comparison to the wild type and empty vector (Fig. 2d). One of the main functions of oleosins in higher plants is to minimize TAG degradation. Thus, it is possible that *AtOLEO3* in Pt4 functions in a similar manner leading to effective TAG accumulation. This is in accordance with the reported overexpression of oleosin in rice kernels and *Arabidopsis* leaves, which both resulted in higher amounts of TAGs [31, 38]. Nonetheless, since gene expression and localization of *AtOLEO3* in LDs of Pt4 was confirmed (Additional file 3: Figure S3; Fig. 2a), we concluded that TAG accumulation in line Pt4O3.1 was a result of *AtOLEO3* expression.

For comparison purposes, the characterization of *ScDGA1* and *AtOLEO3* co-expressing lines (lines Pt4D1O3.1 and Pt4D1O3.2) was done in parallel with the *ScDGA1* expressing line (line Pt4D1.1). We showed that although growth was impaired in the co-expressing lines, TAG accumulation increased by 2.4- and 3.6-fold for Pt4D1O3.1 and Pt4D1O3.2, respectively, in comparison to the wild type (Fig. 3). This increase in TAG levels for both of these lines was higher than a previously reported study for *P. tricornutum* overexpressing the endogenous DGAT2 leading to approximately 1.4-fold increase in the neutral lipid content [37]. Furthermore, co-expressing *ScDGA1* and *AtOLEO3* in Pt4 resulted in significantly higher TAG productivities than when *ScDGA1* was individually expressed (Fig. 3c). In addition, the co-expression of these genes in Pt4 has resulted in a TAG content of 107 µg/mg, which is comparable to the TAG levels observed in the wild type *P. tricornutum* strain Pt1 [39]. In terms of total lipid content, the increase was estimated to be 1.5- and 2.2-folds for Pt4D1O3.1 and Pt4D1O3.2, respectively. Similarly, overexpression of endogenous DGAT2 enzyme in *P. tricornutum* was accompanied by a twofold increase of the total lipid content [40]. This clearly suggests that stacking two genes in an additive manner is more effective than expressing a single gene for improving lipid accumulation in *P.* *tricornutum*. Various other studies have demonstrated the efficiency of such a strategy in other species. For instance, co-expressing DGAT1 and oleosin in *A. thaliana* was more effective in enhancing TAG accumulation in comparison to when DGAT1 was expressed on its own [31]. This also remains in agreement with the role of oleosin in maximizing TAG accumulation also observed in our study. Moreover, in *P. tricornutum*, this double gene strategy has been shown to be effective in modifying the fatty acid composition as the co-expression of a desaturase and an elongase from *O. tauri* had a significant impact on DHA levels in comparison to expressing the desaturase individually [41].

Assessing the impact of *ScDGA1* and *AtOLEO3* co-expression on LD formation in Pt4 showed that high TAG levels in lines Pt4D1O3.1 and Pt4D1O3.2 were accompanied by a formation of very large LDs. This effect was much less obvious in the other lines expressing either the single genes (Pt4D1.1 and Pt4O3.1) or an empty vector (Fig. 4). The formed LDs were less numerous in the co-expressing lines than in the single gene expressing lines but they filled up the majority of the cell volume (Fig. 4c, d). We propose that this is the result of a not yet optimized action of *ScDGA1* and *AtOLEO3*, where *ScDGA1* acts as an efficient TAG provider and oleosin functions as a structural block involved in continuous TAG packaging into enlarging LDs and their prevention from being degraded by lipases. The problem may be solved by expressing the oleosin gene under a stronger promoter than the *ScDGA1* gene. However, similar results were obtained when DGAT1 was co-expressed with oleosin in the leaves of *A. thaliana* resulting in formation of larger LDs when compared to the wild type [31]. Overall, our results showed an evident positive correlation between TAG levels, LD number and sizes.

Since N stress has been commonly shown to enhance TAG accumulation in microalgae [33], we decided to expose the transformants, expressing the transgenes under the control of the fcpA promoter, to N limited conditions. Indeed, under N deprivation, all lines showed an increase in TAG productivities with line Pt4D1O3.2 still showing the highest TAG productivity when compared to other lines (Fig. 5). However, this increase in TAG was accompanied by an increased and early death of the cultured cells leading to an increased sediment of Pt4 cells under these conditions. Overall, this resulted in a significant decrease in biomass rendering this approach unprofitable for Pt4 cultures. Therefore, we suggest that a continuous culture that is continuously harvested may be a preferred strategy for efficient lipid production with Pt4 [24].

We also compared the impact of co-expressing *ScDGA1* and *AtOLEO3* in Pt4 not only under the control of fcpA promoter, but in addition by using the NR promoter. Both, the fcpA and the NR promoter seem to show no differences in TAG productivities under the conditions applied in this study (Fig. 6d). This is an interesting observation since they have been suggested to be active during different growth stages. However, this may be explained again by the assumption that a fast and exponential growth phase may be the primary factor that determines lipid productivity in Pt4.

The fatty acid profile in the TAGs of *P. tricornutum* is dominated by the saturated and monounsaturated fatty acids, with low amounts of VLC-PUFAs like EPA and DHA [17, 28, 41, 42]. In our study, the expression of *ScDGA1* did not result in any modifications in the fatty acid composition and the observed increase in TAG content was likely a result of elevated levels of saturated and monounsaturated fatty acids, which are the most valuable precursor fatty acids for biofuel production. Moreover, the expression of ScDGA1 neither changed the distribution of TAG species nor introduced new TAG molecular species to the TAG pool (Additional file 7: Figure S7). This is in agreement with previous reports [17, 41] and supports the notion that VLC-PUFAs are preferentially channeled into membrane lipids instead of being incorporated into TAGs. This strongly suggests that DGAT activity is not the limiting metabolic step leading to primarily saturated and monounsaturated TAG species [43].

## Conclusions

To our knowledge, we have demonstrated for the first time in microalgae, and especially in a diatom (*P. tricornutum*) that expressing a TAG forming enzyme, yeast DGAT2, together with a plant LD stabilizing and protecting protein, oleosin, has a significant and additive effect on TAG accumulation. An increased TAG productivity was obtained when *ScDGA1* was co-expressed with *AtOLEO3*, in comparison to cultures that expressed *ScDGA1* and *AtOLEO3* as single genes. Interestingly, the fatty acid composition and the profile of TAG molecular species were not altered through the expression of *ScDGA1*, suggesting that although DGAT activity is increasing TAG productivity it is not affecting its composition in this organism. In addition, we have provided first evidence that the expression of a LD protein from plant seeds may protect algal storage lipids from degradation and thereby improve TAG accumulation in microalgae as shown here for *P. tricornutum*. Although N deprivation of the cultures lead to a further increase in TAG productivity, it was accompanied by an increased sedimentation. Thus, rendering N limiting growth conditions unsuitable for optimal lipid production in Pt4. Overall, the approach used in our study was effective at enhancing TAGs species suitable for use in biofuel production. This approach contributes to a promising strategy for enhancing the TAG production in other hosts, including the commercial non-photosynthetic systems such as yeasts since they have much higher productivities than diatoms or microalgae.

## Methods

### Culture conditions for *Phaeodactylum tricornutum*

*Phaeodactylum tricornutum* strain Pt4 was purchased from the SAG culture collection of algae (Göttingen, Germany), with a designation number of SAG 1090-6. Pt4 was cultivated in batch cultures in Erlenmeyer flasks containing f/2 liquid medium [44] was used and its composition was modified by lowering the salt (NaCl) content from 1 to 0.7% (w/v). The cultures were incubated at 20 °C at a speed of 100 rpm, under a 16 h/8 h for day/night cycle, respectively. During the light phase, the cultures were illuminated at a light intensity of 200 µmol m^−2^ s^−1^. Cultures in Erlenmeyer flasks were used as inocula for the inoculation of main cultures in 500 ml glass columns (Ochs Glasgerätebau, Bovenden, Germany). The starting cell concentration for all the experiments was always maintained at 1 × 10^7^ cell/ml. In columns, air supplemented with 1% CO_2_ was used to provide constant aeration to the cultures at a flow rate of 0.15 l/h. Under N deplete conditions, KNO_3_ was omitted from f/2 media. The cells were harvested and washed in N deplete media prior to commencing with the N stress studies in order to ensure complete removal of N. Cell proliferation was assessed through cell concentration, which was measured under a microscope (BX51 Olympus microscope; software, Micro-Manager 1.4.22, Tokyo, Japan) by a Thoma cell counting chamber (Marienfeld, Germany). Chlorophyll a content was measured in order to assess cell proliferation according to a protocol described in [45].

### Cloning of constructs into Pt4

The codon optimized *ScDGA1* and *AtOLEO3* full coding sequences (see Additional file 11) were cloned into Pt4 by the gateway cloning system (Thermo Fischer Scientific, Waltham, USA). Briefly, the *ScDGA1* and A*tOLEO3* genes first cloned into pEntry vectors using the *Spe*I and *Pac*I restriction sites with primers provided in Additional file 12: Table S1. Thereafter, the pEntry vectors were inserted into the pPha-ccdB destination vector, through the LR clonase reaction (Thermo Fischer Scientific, Waltham, USA). The pPha-ccdB destination vector had the pPhaT1 plasmid as a backbone and it consisted of *Sh ble* gene for selection in Zeocin containing f/2 media. Gene expression in Pt4 was driven by the fcpA and NR promoters. The pPha-NR promoter was obtained from Claudia Büchel, (Goethe Universität, Frankfurt am Main, Germany). Pt4 was transformed by particle bombardment according to a protocol described in [46], using a Bio-Rad Biolistic PDS-1000/He particle delivery system fitted with 1350 psi rupture disks. Following bombardment, cells were left to recover by incubation at 20 °C for 24 h in f/2 agar plates without a selection marker. Thereafter, they were transferred to f/2 agar plates containing zeocin (75 µg/ml). The surviving lines were transferred into fresh selection liquid f/2 media contained in microtitre plates for further characterization.

### Analysis of transgenic Pt4

The selection criterion for transformants during the first screening of transgenic Pt4 was the expression of the transgene. This involved a random selection of transformants (each transformation round resulted in ~100 transformants of which 50 were tested for transgene integration, thereafter 10 transformants positive for transgene integration were tested for transgene expression. Transformants positive for transgene integration were then further checked for transgene expression, of which positive transformants were further processed for lipid yields. Genomic DNA was extracted using a Cetyltrimethyl Ammonium Bromide (CTAB) buffer (2% CTAB, 10 mM Tris–HCl pH 8, 20 mM EDTA pH 8, 1.4 M NaCl). The extracted genomic DNA then served as templates for amplification of *ScDGA*1 and *AtOLEO3*, using primers provided in Additional file 13: Table S2. Pt4 lines positive for gene integration were further analyzed for gene expression, which was done by semi-quantitative PCR. Briefly, RNA was extracted from fresh biomass using TRIZOL buffer according to a protocol described in [47]. The extracted RNA was treated with DNase I enzyme (Thermo Fischer Scientific, Waltham, USA) prior to cDNA synthesis, which was done by the RevertAid H-minus reverse transcriptase (Thermo Fischer Scientific, Waltham, USA), according to the manufacturer’s instructions. Standard PCR were then carried out to amplify the *ScDGA1* and *AtOLEO3* genes from the cDNA generated, using the primers provided in Additional file 13: Table S2. Lines that were positive for gene expression were bulked up for the analysis of growth and lipid accumulation.

### Extraction and analysis of lipids from Pt4

Total lipids were extracted from lyophilized biomass with modifications according to a protocol described in [48]. Briefly, total lipids were extracted from 10 mg of lyophilized biomass using MTBE as the organic solvent. Prior to lipid extraction, internal standards were added; these were tri-15:0 TAG (Sigma-Aldrich, Munich, Germany) and Di-17:0 PC (Avanti Polar Lipids, Alabama, USA). TAGs were extracted from the total lipids by thin layer chromatography (TLC) using hexane:diethyl ether:acetic (80:20:1, v/v/v) acid as a mobile solvent. TAG bands were visualized by first spraying the TLC places with 0.2% (w/v) 8-anilinonaphthalene-1-sulfonic acid followed by exposure to UV light. TAG bands were scraped out from the TLC plates for further analysis. Total lipid and TAG samples were transesterified to generate fatty acid methyl esters (FAMEs) according to a protocol described in [49], however in the presence of an acid (H_2_SO_4_) instead of a base. FAMEs generated were subsequently analyzed by gas chromatography (Agilent GC 6890, Agilent Technologies, Waldbroon, Germany) coupled to a flame ionization detector (GC-FID). Samples were injected at temperature of 220 °C and a volume of 1 µl using a split mode injection. The separation of fatty acids was carried out in a DB-23 column (30 m × 0.25 mm with 0.25 µm coating thickness; Agilent Technologies, Waldbroon, Germany). Helium was used as a carrier gas and the temperature gradient used was 150 °C for 1 min, 150–200 °C at 4 °C/min, 200–250 °C at 20 °C/min, and 250 °C for 3 min. A FAME mix (C4–C24, Sigma-Aldrich, Munich, Germany) was used an external standard for fatty acid identification. The signals were integrated using the ChemStation Software (Agilent Technologies, Santa Clara, USA).

### Analysis of TAG molecular species by UPLC-nano ESI-MS/MS

Total lipid extraction for this analysis was carried out in a similar manner as described above. The final lipid extracts were re-suspended in 0.8 ml tetrahydrofuran:methanol:water (4:4:1, v/v/v) prior to analysis. The separation of TAG species from the total extract was carried with a UPLC-ESI-MS/MS system (Waters Corp., Milford, USA) according to a protocol described in [50]. The system was equipped with the ACQUITY UPLC HSS T3 column (100 mm × 1 mm, 1.8 µm; Waters Corp., USA). Samples were injected at a volume of 2 µl, using the needle overfill mode. The flow rate was set at 0.10 ml/min with a separation temperature of 35 °C.

### Extraction and analysis of acyl-CoAs

Acyl-CoAs were extracted from the highest TAG accumulating line (Pt4D1O3.2) grown under N replete conditions as well as from the empty vector and wild type according to a protocol described in [51], with some modifications. Briefly, 20 mg of lyophilized biomass was homogenized in 200 µl of freshly prepared extraction buffer [2-propanol, 2 ml; 50 mM KH_2_PO_4_ pH 7.2, 2 ml; acetic acid, 50 µl and 50 mg/ml BSA (fatty acid free), 80 µl]. The homogenate was mixed with 300 µl of saturated petroleum ether (saturated with 2-propanol: water (1:1, v/v). To force phase separation, samples were spun at 500 *g* or 2 min and the interphase was collected and washed with the saturated petroleum ether three times. Following the washing steps, the interphase fractions were mixed with 5 µl of saturated (NH_4_)_2_SO_4_ and 600 µl of methanol:chloroform (2:1, v/v). Samples were then mixed thoroughly prior to incubation at room temperature for 20 min. Thereafter, they were centrifuged at 21,000*g* for 2 min. The supernatant was collected and dried under a stream of nitrogen and re-suspended in 300 µl of derivatization buffer [0.5 M chloracetaldehyde; 0.15 M citrate buffer, pH4, and 0.5% (w/v) SDS], and derivatized at 85 °C for 20 min. Thereafter, samples were cooled down and analyzed by high-performance liquid chromatography (HPLC), as described [51].

### Assessment of the morphology and number of LDs

Cells were fixed in a mixture of 4% (w/v) paraformaldehyde in PBS (pH 7.4) overnight at 4 °C. After three washes in PBS buffer, cells were incubated with Bodipy 493/504 (Thermo Scientific, Grand Island, USA) at a final concentration of 10 µg/ml in PBS buffer for 1 h at room temperature. This was followed by the washing of cells three times in the same buffer and re-suspended in ProLong Gold anti-fade reagent (Thermo Fischer Scientific, Waltham, USA). Samples were then analyzed with a Zeiss LSM 510 META confocal laser scanning microscope (Carl Zeiss, Jena, Germany) using a 63× Plan-Apochromat 1.4 NA oil-immersion lens. An argon laser was used for Bodipy 493/504 and chlorophyll excitation and the emission spectra were collected in two separate channels, 500–515 nm and 630–670 nm, respectively. *Z*-series images were collected and processed with the LSM 5 Image Browser (Carl Zeiss, Jena, Germany). LD morphometrics were performed by the same software using 3D reconstruction confocal images of 70 cells for each analyzed line.

## Additional files

**Additional file 1: Figure S1.** Semi-quantitative PCR for *ScDGA1* expressing lines. Screening for lines expressing the *ScDGA1* gene was done by semi-quantitative PCR where the cDNA was used as a template. cDNA extracted from the wild type was used as a negative control. Lines highlighted in red were selected for further analysis. M = marker, nc = negative control.

**Additional file 2: Figure S2.** The impact of *ScDGA1* on the FA composition in the total lipids (a) and TAGs (b) of Pt4. The FA composition is presented in relative amounts (% contribution of each FA). The mean values on the bars represent values from two independent studies, where in each study there were two biological replicates. This data is representative of samples taken on day 7 of culture, where maximum yields of lipids were accumulated. EV = empty vector, FA = fatty acid, TAG = triacylglycerol, WT = wild type. Raw data for fatty acid composition can be obtained from Additional file 14.

**Additional file 3: Figure S3.** Semi-quantitative PCR for *AtOLEO3* expressing lines. Screening for lines expressing the *AtOLEO3* gene was by semi-quantitative PCR where the cDNA was used as a template. cDNA extracted from the wild type was used as a negative control. Lines highlighted in red were selected for further analysis. M = marker, nc = negative control.

**Additional file 4: Figure S4.** The effect of *AtOLEO3* expression on the FA composition in the total lipids (a) and TAGs (b) of Pt4. The FA composition is presented in relative amounts (% contribution of each FA). Data points on the bars represent mean values from three independent studies, where in each study there were two biological replicates. Error bars were calculated from the standard deviation. This data is representative of samples taken on day 14 of culture, where maximum yields of lipids were accumulated. EV = empty vector, FA = fatty acid, TAG = triacylglycerol, WT = wild type. Raw data for fatty acid composition can be obtained from Additional file 14.

**Additional file 5: Figure S5.** Screening for lines co-expressing the *ScDGA1* and *AtOLEO3* genes. This screening was carried out by semi-quantitative PCR where the cDNA was used as a template. cDNA extracted from the wild type was used as a negative control. Lines highlighted in red were selected for further analysis. M = marker, nc = negative control.

**Additional file 6: Figure S6.** The impact of *ScDGA1* and *AtOLEO3* co-expression on the FA composition in the total lipids (a) and TAGs (b) of Pt4. The FA composition is presented in relative amounts (% contribution of each FA). Data points on the bars represent mean value from three independent studies, where in each study there were two biological replicates. Error bars were calculated from the standard deviation. This data is representative of samples taken on day 17. EV = empty vector, FA = fatty acid, TAG = triacylglycerol, WT = wild type. Raw data for fatty acid composition can be obtained from Additional file 14.

**Additional file 7: Figure S7.** The effect of ScDGA1 on the distribution of TAG molecular species. The distribution of TAG molecular species is presented in relative amounts (b) and the species were summed up to indicate the difference in TAG accumulation between transformants and controls (a). These bar graphs represent mean values of two biological replicates from a single experiment. The samples were taken from day 21 of culture, the last day in a growth curve. EV = empty vector, TAG = triacylglycerol, WT = wild type. Raw data for TAG molecular species can be obtained from Additional file 14.

**Additional file 8: Figure S8.** Acyl-CoA pool composition in the multigene expressing lines and controls. The acyl-CoAs were extracted from the highest TAG accumulating line (Pt4D1O3.2), empty vector control (EV) and from the wild type strain of Pt4 (WT) on the last day (21st) of the growth curves. Error bars were calculated from the standard deviation, with three technical replicates from a single experiments. EV = empty vector, WT = wild type. Raw data for acyl-CoA pool composition can be obtained from Additional file 14.

**Additional file 9: Figure S9.** Comparison of the fcpA and NR promoters in driving YFP expression in Pt4. In both lines, the YFP signal is present in the cytosol. The EV line was used as a negative control and it showed no YFP fluorescence. Images are composites of BF, YFP fluorescence (green), chlorophyll autofluorescence (red) and merged images. Scale bar = 5 µm. BF = bright field, EV = empty vector, NR = nitrate reductase, YFP = yellow fluorescent protein.

**Additional file 10: Figure S10.** Semi-quantitative PCR for *ScDGA1* and *AtOLEO3* co-expressing lines under the fcpA promoter and NR promoter. Screening for lines co-expressing the *ScDGA1* and *AtOLEO3* genes was by semi-quantitative PCR where the cDNA was used as a template. cDNA extracted from the wild type was used as a negative control. M = marker, nc = negative control, NR = nitrate reductase.

**Additional file 11.** Nucleotide sequences for *ScDGA1* and *AtOLEO3* used in this study.

**Additional file 12: Table S1.** Restriction site primers used for the cloning of *ScDGA1* and AtOLEO3.

**Additional file 13: Table S2.** Primers used for assessing gene integration and expression of *ScDGA1* and *AtOLEO3* genes in Pt4.

**Additional file 14.** Raw data used to generate the figures. Data used to generate Fig. 1 and Additional file 2: Figure S2 is presented under tab, ScDGA1. Raw data for Fig. 2 and Additional file 4: Figure S4 is presented under tab, AtOLEO3. Raw data for Fig. 3 and Additional file 6: Figure S6 is presented under tab, ScDGA1 + AtOLEO3. Raw data for Fig. 4 is presented under tab LD morphology. Raw data for Fig. 5 is presented under tab, ScDGA1 + AtOLEO3 N deplete. Raw data for Fig. 6 is presented under tab ScDGA1 + AtOLEO3 NR promoter. Raw data for Additional file 7: Figure S7 is presented under tab, TAG molecular species. Raw data for Additional file 8: Figure S8 is presented under tab Acyl-CoA pool.

## Abbreviations

AtOLEO3: oleosin 3 from *Arabidopsis thaliana*
CLSM: confocal laser scanning microscopy
DGAT: acyl-CoA:diacylglycerol acyltransferase
FA: fatty acid
FAME: fatty acid methyl ester
GC-FID: gas chromatography-flame ionization detector
HPLC: high-performance liquid chromatography
LD: lipid droplet
ScDGA1: acyl-CoA:diacylglycerol acyltransferase 1 from *Saccharomyces cerevisiae*
TAG: triacylglycerol
UPLC: ultra-performance liquid chromatography
VLC-PUFA: very long-chain polyunsaturated fatty acid

## References

[1] Yao Y, Lu Y, Peng K-T, Huang T, Niu Y-F, Xie W-H, Yang W-D, Liu J-S, Li H-Y (2014). Glycerol and neutral lipid production in the oleaginous marine diatom *Phaeodactylum tricornutum* promoted by overexpression of glycerol-3-phosphate dehydrogenase. Biotechnol Biofuels.

[2] Ruiz J, Olivieri G, de Vree J, Bosma R, Willems P, Reith JH, Eppink MHM, Kleinegris DMM, Wijffels RH, Barbosa MJ (2016). Towards industrial products from microalgae. Energy Environ Sci..

[3] Hamilton ML, Warwick J, Terry A, Allen MJ, Napier JA, Sayanova O (2015). Towards the industrial production of omega-3 long chain polyunsaturated fatty acids from a genetically modified diatom *Phaeodactylum tricornutum*. PLoS ONE.

[4] Khozin-Goldberg I, Leu S, Boussiba S, Nakamura Y, Li-Beisson Y (2016). Microalgae as a source for VLC-PUFA production. Lipids in plant and algae development.

[5] Li-Beisson Y, Shorrosh B, Beisson F, Andersson MX, Arondel V, Bates PD, Baud S, Bird D, DeBono A, Durrett TP et al. Acyl-lipid metabolism. In: The arabidopsis book. The American Society of Plant Biologists; 2013. p. e0161. 10.1199/tab.0161 PMC3563272 23505340

[6] Hannon M, Gimpel J, Tran M, Rasala B, Mayfield S (2010). Biofuels from algae: challenges and potential. Biofuels..

[7] Medipally SR, Yusoff FM, Banerjee S, Shariff M (2015). Microalgae as sustainable renewable energy feedstock for biofuel production. BioMed Res Int.

[8] Du Z-Y, Benning C, Nakamura Y, Li-Beisson Y (2016). Triacylglycerol accumulation in photosynthetic cells in plants and algae. Lipids in Plant and Algae Development.

[9] Dong T, Knoshaug EP, Pienkos PT, Laurens LML (2016). Lipid recovery from wet oleaginous microbial biomass for biofuel production: a critical review. Appl Energy.

[10] Cao H (2011). Structure-function analysis of diacylglycerol acyltransferase sequences from 70 organisms. BMC Res Notes..

[11] Chapman KD, Ohlrogge JB (2012). Compartmentation of triacylglycerol accumulation in plants. J Biol Chem.

[12] Cagliari A, Margis R, dos Santos Maraschin F, Turchetto-Zolet AC, Loss G, Margis-Pinheiro M (2011). Biosynthesis of triacylglycerols (TAGs) in plants and algae. Int J Plant Biol..

[13] Sorger D, Daum G (2003). Triacylglycerol biosynthesis in yeast. Appl Microbiol Biotechnol.

[14] Wagner M, Hoppe K, Czabany T, Heilmann M, Daum G, Feussner I, Fulda M (2010). Identification and characterization of an acyl-CoA:diacylglycerol acyltransferase 2 (DGAT2) gene from the microalga *O. tauri*. Plant Physiol Biochem.

[15] Zienkiewicz K, Du Z-Y, Ma W, Vollheyde K, Benning C (2016). -induced neutral lipid biosynthesis in microalgae—molecular, cellular and physiological insights. Biochim Biophys Acta.

[16] Zienkiewicz K, Zienkiewicz A, Poliner E, Du Z-Y, Vollheyde K, Herrfurth C, Marmon S, Farré EM, Feussner I, Benning C (2017). Nannochloropsis, a rich source of diacylglycerol acyltransferases for engineering of triacylglycerol content in different hosts. Biotechnol Biofuels.

[17] Popko J, Herrfurth C, Feussner K, Ischebeck T, Iven T, Haslam R, Hamilton M, Sayanova O, Napier J, Khozin-Goldberg I (2016). Metabolome analysis reveals betaine lipids as major source for triglyceride formation, and the accumulation of sedoheptulose during nitrogen-starvation of *Phaeodactylum tricornutum*. PLoS ONE.

[18] Chapman KD, Dyer JM, Mullen RT (2012). Biogenesis and functions of lipid droplets in plants. J Lipid Res.

[19] Huang M-D, Huang AHC (2015). Bioinformatics reveal five lineages of oleosins and the mechanism of lineage evolution related to structure/function from green algae to seed plants. Plant Physiol.

[20] Yoneda K, Yoshida M, Suzuki I, Watanabe MM (2016). Identification of a major lipid droplet protein in a marine diatom *Phaeodactylum tricornutum*. Plant Cell Physiol.

[21] Huang N-L, Huang M-D, Chen T-LL, Huang AHC (2013). Oleosin of subcellular lipid droplets evolved in green algae. Plant Physiol.

[22] Moellering ER, Benning C (2010). RNA interference silencing of a major lipid droplet protein affects lipid droplet size in *Chlamydomonas reinhardtii*. Eukaryot Cell.

[23] Vieler A, Brubaker SB, Vick B, Benning C (2012). A lipid droplet protein of Nannochloropsis with functions partially analogous to plant oleosins. Plant Physiol.

[24] Vinayak V, Manoylov KM, Gateau H, Blanckaert V, Hérault J, Pencréac’h G, Marchand J, Gordon R, Schoefs B (2015). Diatom milking: a review and new approaches. Mar Drugs..

[25] Merz CR, Main KL. Microalgae (diatom) production-the aquaculture and biofuel nexus. In: 2014 Oceans-St John’s. St John’s: IEEE; 2014. p. 1–10.

[26] Fu W, Wichuk K, Brynjólfsson S (2015). Developing diatoms for value-added products: challenges and opportunities. New Biotechnol.

[27] Vílchez C, Forján E, Cuaresma M, Bédmar F, Garbayo I, Vega JM (2011). Marine carotenoids: biological functions and commercial applications. Mar Drugs..

[28] Abida H, Dolch L-J, Mei C, Villanova V, Conte M, Block MA, Finazzi G, Bastien O, Tirichine L, Bowler C (2015). Membrane glycerolipid remodeling triggered by nitrogen and phosphorus starvation in *Phaeodactylum tricornutum*. Plant Physiol.

[29] Guihéneuf F, Leu S, Zarka A, Khozin-Goldberg I, Khalilov I, Boussiba S (2011). Cloning and molecular characterization of a novel acyl-CoA:diacylglycerol acyltransferase 1-like gene (PtDGAT1) from the diatom *Phaeodactylum* *tricornutum*. FEBS J.

[30] Zaslavskaia LA, Lippmeier JC, Kroth PG, Grossman AR, Apt KE (2000). Transformation of the diatom *Phaeodactylum tricornutum* (Bacillariophyceae) with a variety of selectable marker and reporter genes. J Phycol.

[31] Winichayakul S, Scott RW, Roldan M, Hatier J-HB, Livingston S, Cookson R, Curran AC, Roberts NJ (2013). In vivo packaging of triacylglycerols enhances Arabidopsis leaf biomass and energy density. Plant Physiol.

[32] Vanhercke T, El Tahchy A, Liu Q, Zhou X-R, Shrestha P, Divi UK, Ral J-P, Mansour MP, Nichols PD, James CN (2014). Metabolic engineering of biomass for high energy density: oilseed-like triacylglycerol yields from plant leaves. Plant Biotechnol J.

[33] Li-Beisson Y, Nakamura Y, Harwood J, Nakamura Y, Li-Beisson Y (2016). Lipids: from chemical structures, biosynthesis, and analyses to industrial applications. Lipids in plant and algae development.

[34] Chu L, Ewe D, Río Bártulos C, Kroth PG, Gruber A (2016). Rapid induction of GFP expression by the nitrate reductase promoter in the diatom *Phaeodactylum tricornutum*. PeerJ..

[35] Kamisaka Y, Kimura K, Uemura H, Yamaoka M (2013). Overexpression of the active diacylglycerol acyltransferase variant transforms *Saccharomyces cerevisiae* into an oleaginous yeast. Appl Microbiol Biotechnol.

[36] Tan KWM, Lee YK (2016). The dilemma for lipid productivity in green microalgae: importance of substrate provision in improving oil yield without sacrificing growth. Biotechnol Biofuels.

[37] Niu Y-F, Zhang M-H, Li D-W, Yang W-D, Liu J-S, Bai W-B, Li H-Y (2013). Improvement of neutral lipid and polyunsaturated fatty acid biosynthesis by overexpressing a type 2 diacylglycerol acyltransferase in marine diatom *Phaeodactylum tricornutum*. Mar Drugs..

[38] Wu Y-Y, Chou Y-R, Wang C-S, Tseng T-H, Chen L-J, Tzen JTC (2010). Different effects on triacylglycerol packaging to oil bodies in transgenic rice seeds by specifically eliminating one of their two oleosin isoforms. Plant Physiol Biochem.

[39] Lu Y, Wang X, Balamurugan S, Yang W-D, Liu J-S, Dong H-P, Li H-Y (2017). Identification of a putative seipin ortholog involved in lipid accumulation in marine microalga *Phaeodactylum tricornutum*. J Appl Phycol..

[40] Dinamarca J, Levitan O, Kumaraswamy GK, Lun DS, Falkowski PG (2017). Overexpression of a diacylglycerol acyltransferase gene in *Phaeodactylum tricornutum* directs carbon towards lipid biosynthesis. J Phycol.

[41] Hamilton ML, Haslam RP, Napier JA, Sayanova O (2014). Metabolic engineering of *Phaeodactylum tricornutum* for the enhanced accumulation of omega-3 long chain polyunsaturated fatty acids. Metabol Eng..

[42] Mühlroth A, Li K, Røkke G, Winge P, Olsen Y, Hohmann-Marriott M, Vadstein O, Bones A (2013). Pathways of lipid metabolism in marine algae, co-expression network, bottlenecks and candidate genes for enhanced production of EPA and DHA in species of Chromista. Mar Drugs..

[43] Bigogno C, Khozin-Goldberg I, Adlerstein D, Cohen Z (2002). Biosynthesis of arachidonic acid in the oleaginous microalga *Parietochloris incisa* (*Chlorophyceae*): radiolabeling studies. Lipids.

[44] Guillard RR, Ryther JH (1962). Studies of marine planktonic diatoms. I. *Cyclotella nana* Hustedt, and *Detonula confervacea* (cleve) Gran. Can J Microbiol.

[45] Khozin-Goldberg I, Shrestha P, Cohen Z (2005). Mobilization of arachidonyl moieties from triacylglycerols into chloroplastic lipids following recovery from nitrogen starvation of the microalga *Parietochloris incisa*. Biochim Biophys Acta.

[46] Falciatore A, Casotti R, Leblanc C, Abrescia C, Bowler C (1999). Transformation of nonselectable reporter genes in marine diatoms. Mar Biotechnol.

[47] Chomczynski P, Sacchi N (1987). Single-step method of RNA isolation by acid guanidinium thiocyanate-phenol-chloroform extraction. Anal Biochem.

[48] Matyash V, Liebisch G, Kurzchalia TV, Shevchenko A, Schwudke D (2008). Lipid extraction by methyl-tert-butyl ether for high-throughput lipidomics. J Lipid Res.

[49] Hornung E, Korfei M, Pernstich C, Struss A, Kindl H, Fulda M, Feussner I (2005). Specific formation of arachidonic acid and eicosapentaenoic acid by a front-end D^5^-desaturase from *Phytophthora megasperma*. Biochim Biophys Acta.

[50] Tarazona P, Feussner K, Feussner I (2015). An enhanced plant lipidomics method based on multiplexed liquid chromatography–mass spectrometry reveals additional insights into cold- and drought-induced membrane remodeling. Plant J..

[51] Larson TR, Graham IA (2001). Technical Advance: a novel technique for the sensitive quantification of acyl CoA esters from plant tissues. Plant J..

