# Supplementary figures and images for: Heterologous co-expression of a yeast diacylglycerol acyltransferase (ScDGA1) and a plant oleosin (AtOLEO3) as an efficient tool for enhancing triacylglycerol accumulation in the marine diatom Phaeodactylum tricornutum
Source: Biotechnol Biofuels. 2017 Jul 17;10:187. doi: 10.1186/s13068-017-0874-1 (PMC5514505; doi:10.1186/s13068-017-0874-1)

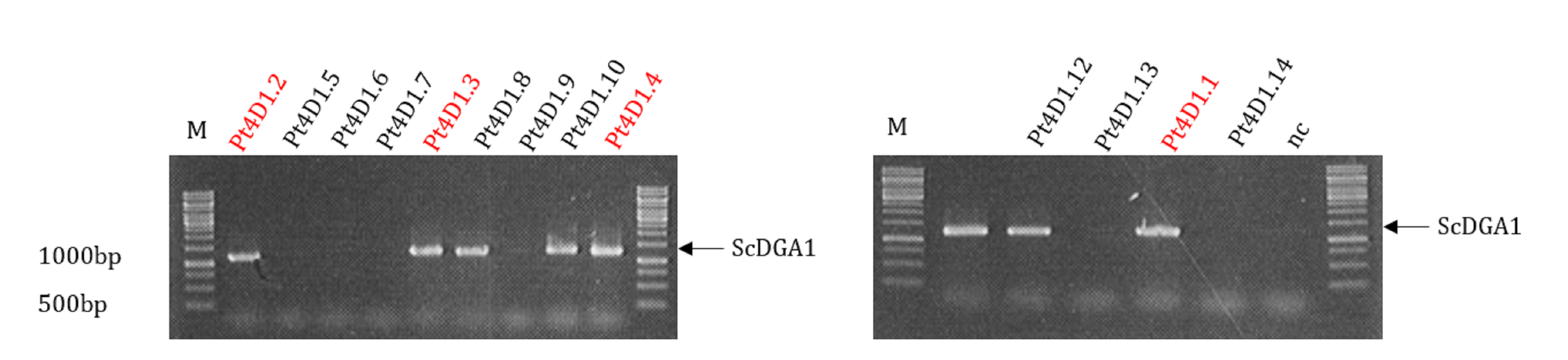

Supplement: Supplementary file 1 — Additional file 1: Figure S1. Semi-quantitative PCR for ScDGA1 expressing lines. Screening for lines expressing the ScDGA1 gene was done by semi-quantitative PCR where the cDNA was used as a template. cDNA extracted from the wild type was used as a negative control. Lines highlighted in red were selected for further analysis. M = marker, nc = negative control. [file 13068_2017_874_MOESM1_ESM.tif]

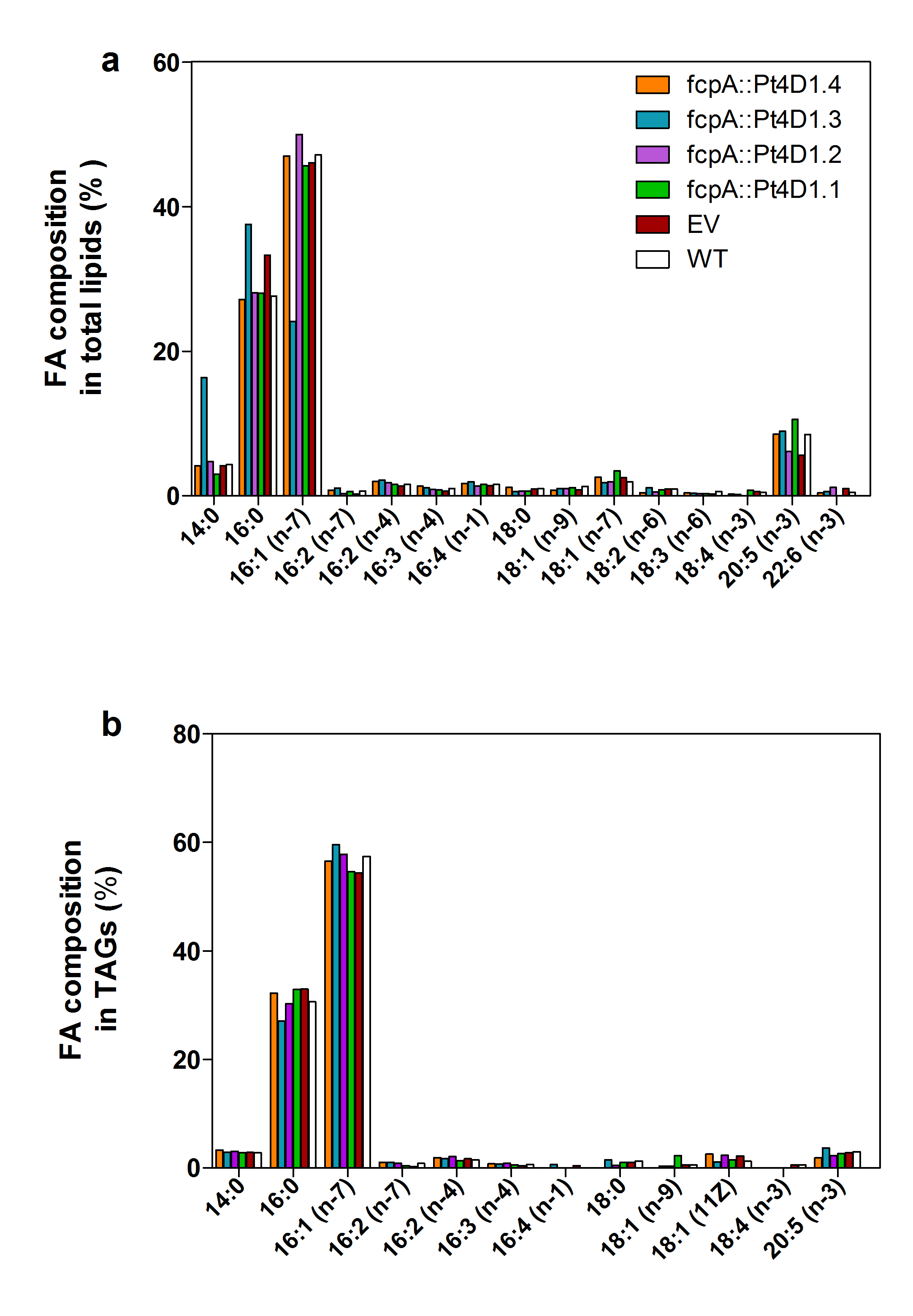

Supplement: Supplementary file 2 — Additional file 2: Figure S2. The impact of ScDGA1 on the FA composition in the total lipids (a) and TAGs (b) of Pt4. The FA composition is presented in relative amounts (% contribution of each FA). The mean values on the bars represent values from two independent studies, where in each study there were two biological replicates. This data is representative of samples taken on day 7 of culture, where maximum yields of lipids were accumulated. EV = empty vector, FA = fatty acid, TAG = triacylglycerol, WT = wild type. Raw data for fatty acid composition can be obtained from Additional file 14. [file 13068_2017_874_MOESM2_ESM.tif]

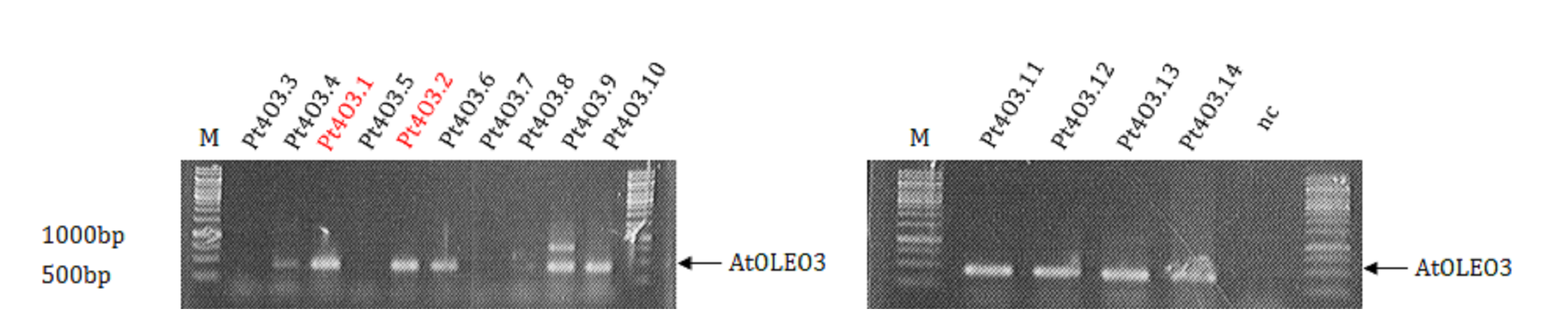

Supplement: Supplementary file 3 — Additional file 3: Figure S3. Semi-quantitative PCR for AtOLEO3 expressing lines. Screening for lines expressing the AtOLEO3 gene was by semi-quantitative PCR where the cDNA was used as a template. cDNA extracted from the wild type was used as a negative control. Lines highlighted in red were selected for further analysis. M = marker, nc = negative control. [file 13068_2017_874_MOESM3_ESM.tif]

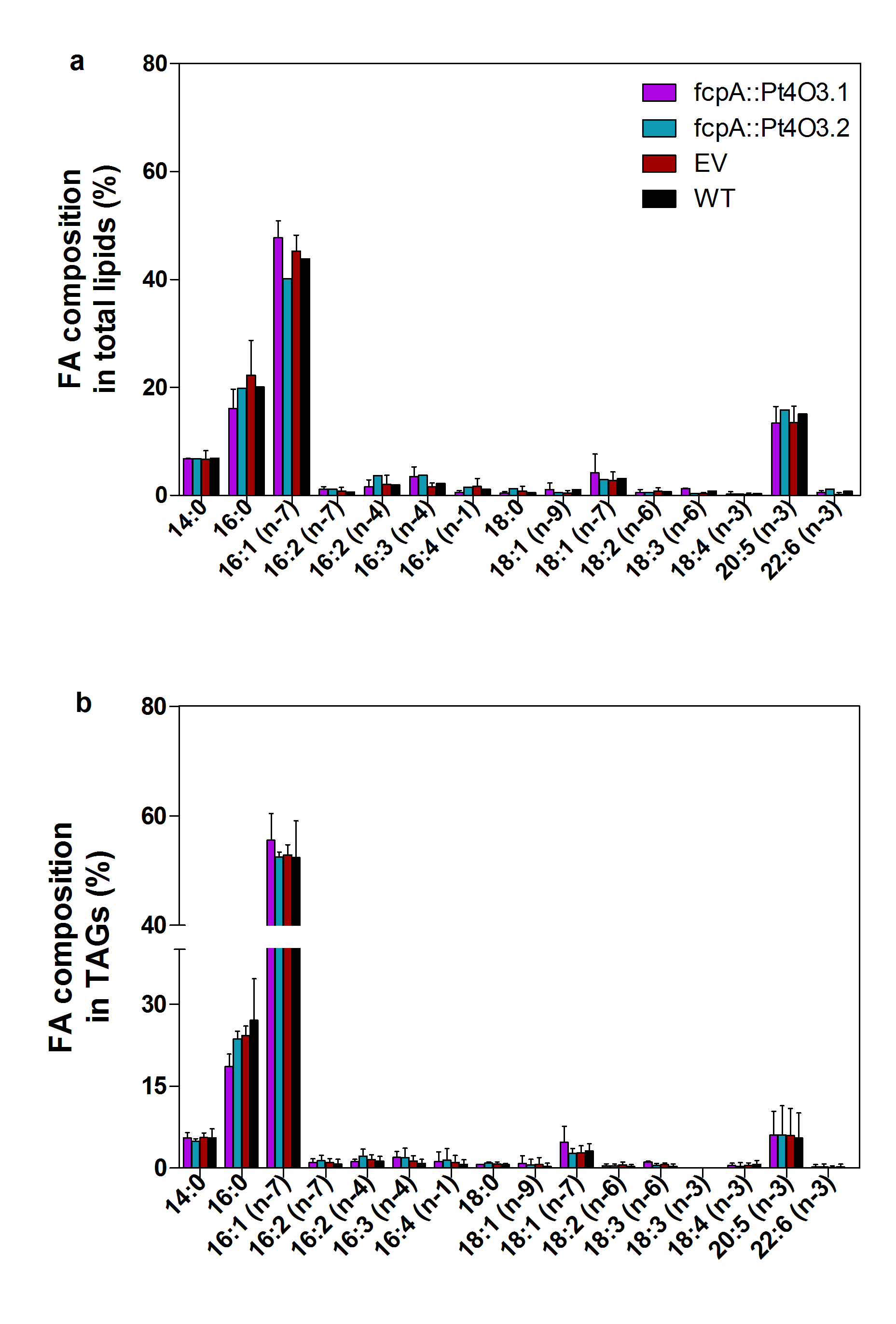

Supplement: Supplementary file 4 — Additional file 4: Figure S4. The effect of AtOLEO3 expression on the FA composition in the total lipids (a) and TAGs (b) of Pt4. The FA composition is presented in relative amounts (% contribution of each FA). Data points on the bars represent mean values from three independent studies, where in each study there were two biological replicates. Error bars were calculated from the standard deviation. This data is representative of samples taken on day 14 of culture, where maximum yields of lipids were accumulated. EV = empty vector, FA = fatty acid, TAG = triacylglycerol, WT = wild type. Raw data for fatty acid composition can be obtained from Additional file 14. [file 13068_2017_874_MOESM4_ESM.tif]

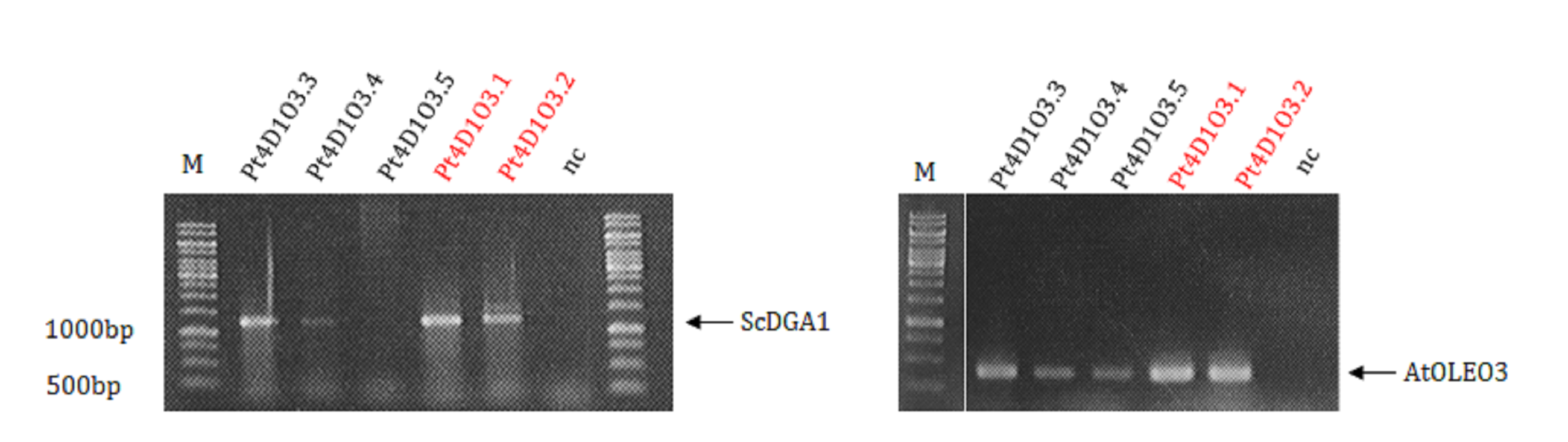

Supplement: Supplementary file 5 — Additional file 5: Figure S5. Screening for lines co-expressing the ScDGA1 and AtOLEO3 genes. This screening was carried out by semi-quantitative PCR where the cDNA was used as a template. cDNA extracted from the wild type was used as a negative control. Lines highlighted in red were selected for further analysis. M = marker, nc = negative control. [file 13068_2017_874_MOESM5_ESM.tif]

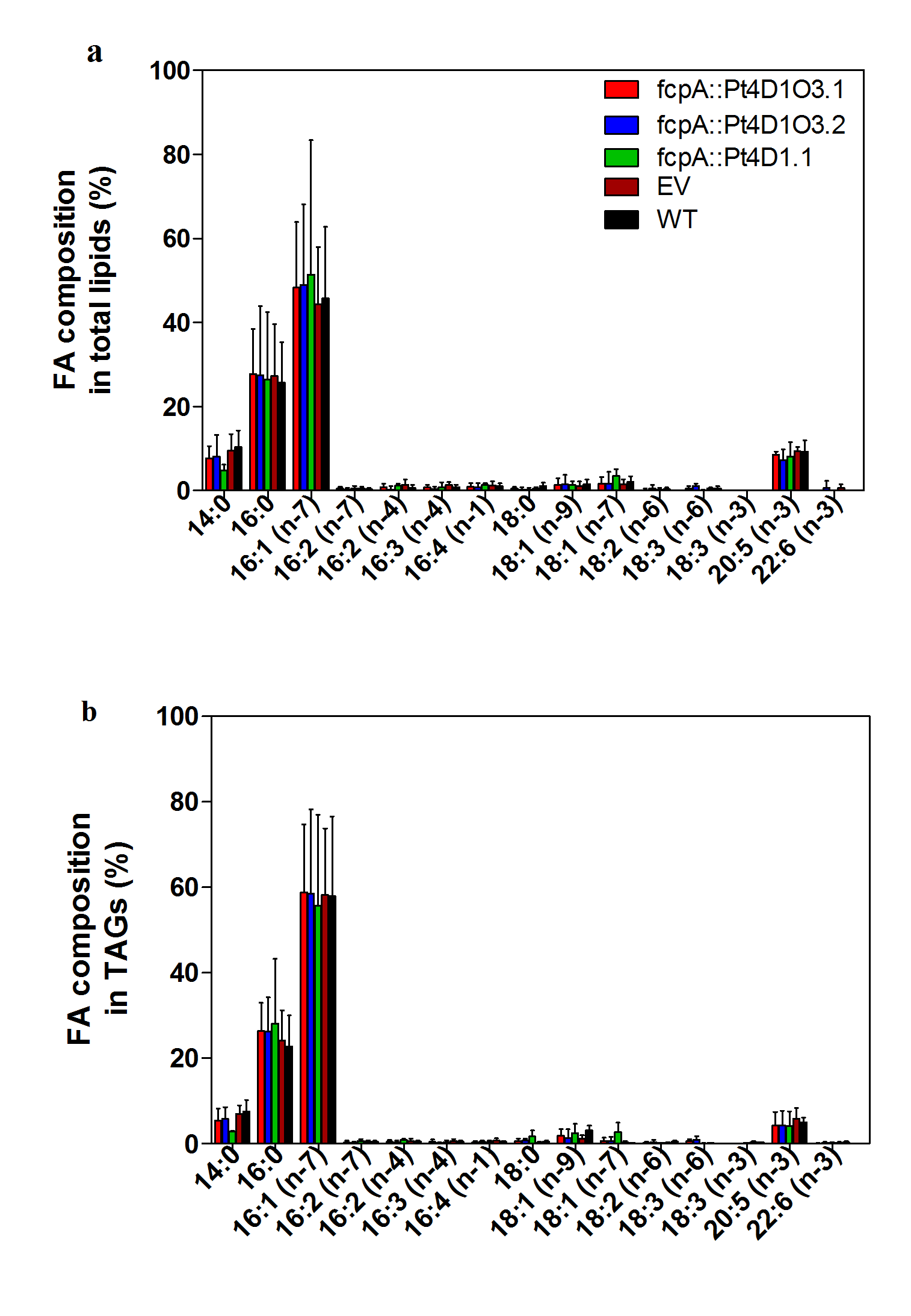

Supplement: Supplementary file 6 — Additional file 6: Figure S6. The impact of ScDGA1 and AtOLEO3 co-expression on the FA composition in the total lipids (a) and TAGs (b) of Pt4. The FA composition is presented in relative amounts (% contribution of each FA). Data points on the bars represent mean value from three independent studies, where in each study there were two biological replicates. Error bars were calculated from the standard deviation. This data is representative of samples taken on day 17. EV = empty vector, FA = fatty acid, TAG = triacylglycerol, WT = wild type. Raw data for fatty acid composition can be obtained from Additional file 14. [file 13068_2017_874_MOESM6_ESM.tif]

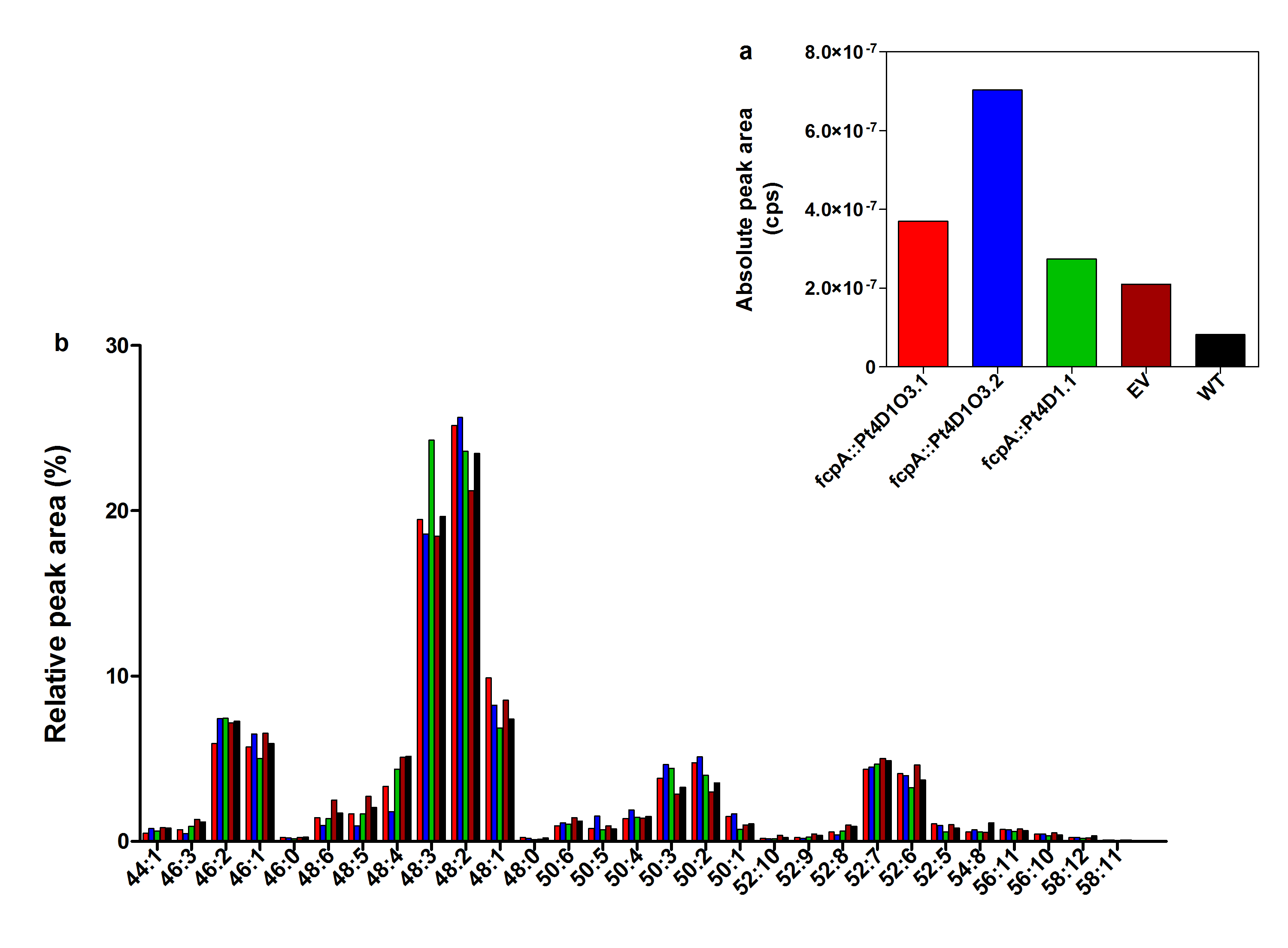

Supplement: Supplementary file 7 — Additional file 7: Figure S7. The effect of ScDGA1 on the distribution of TAG molecular species. The distribution of TAG molecular species is presented in relative amounts (b) and the species were summed up to indicate the difference in TAG accumulation between transformants and controls (a). These bar graphs represent mean values of two biological replicates from a single experiment. The samples were taken from day 21 of culture, the last day in a growth curve. EV = empty vector, TAG = triacylglycerol, WT = wild type. Raw data for TAG molecular species can be obtained from Additional file 14. [file 13068_2017_874_MOESM7_ESM.tif]

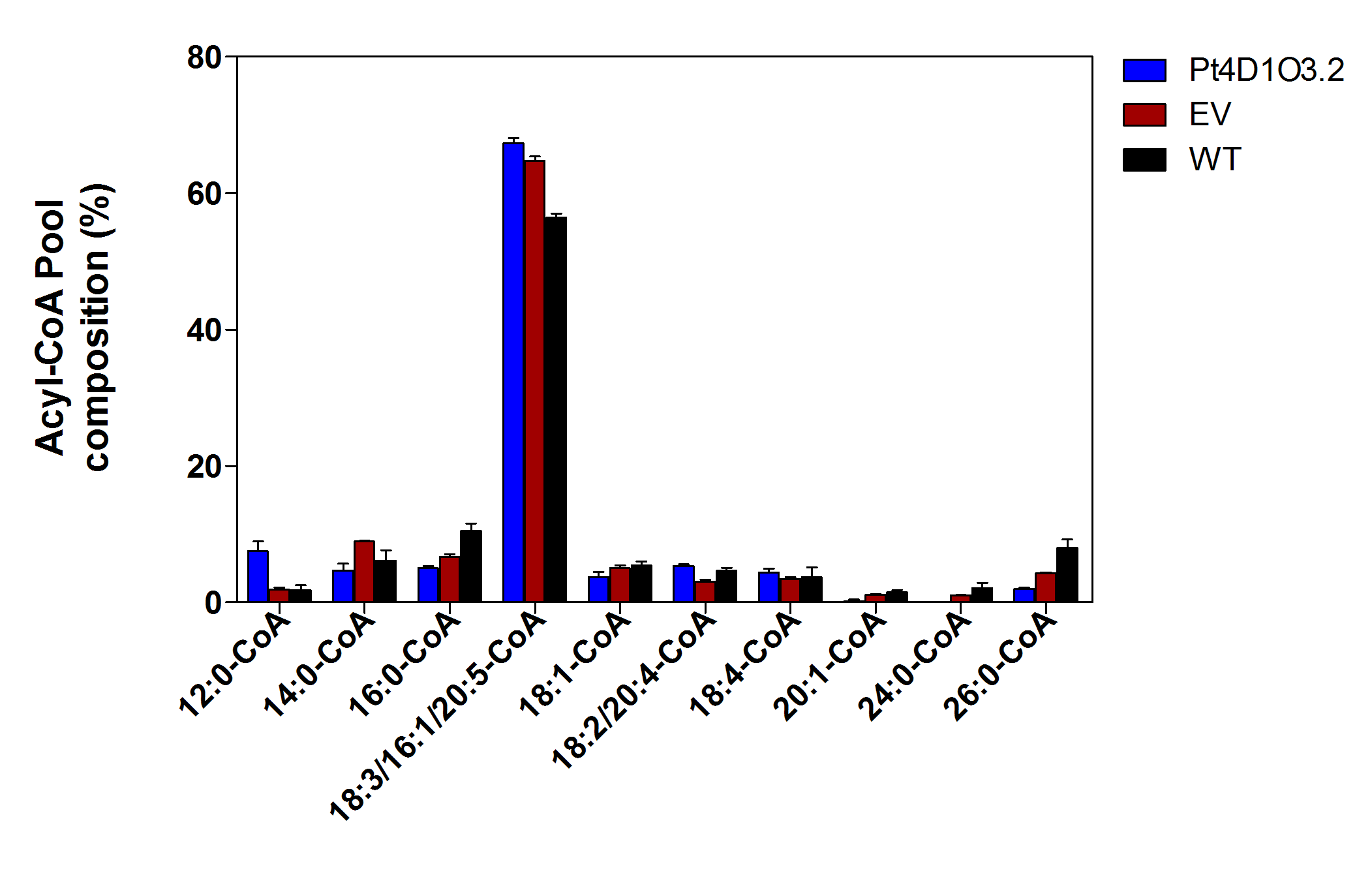

Supplement: Supplementary file 8 — Additional file 8: Figure S8. Acyl-CoA pool composition in the multigene expressing lines and controls. The acyl-CoAs were extracted from the highest TAG accumulating line (Pt4D1O3.2), empty vector control (EV) and from the wild type strain of Pt4 (WT) on the last day (21st) of the growth curves. Error bars were calculated from the standard deviation, with three technical replicates from a single experiments. EV = empty vector, WT = wild type. Raw data for acyl-CoA pool composition can be obtained from Additional file 14. [file 13068_2017_874_MOESM8_ESM.tif]

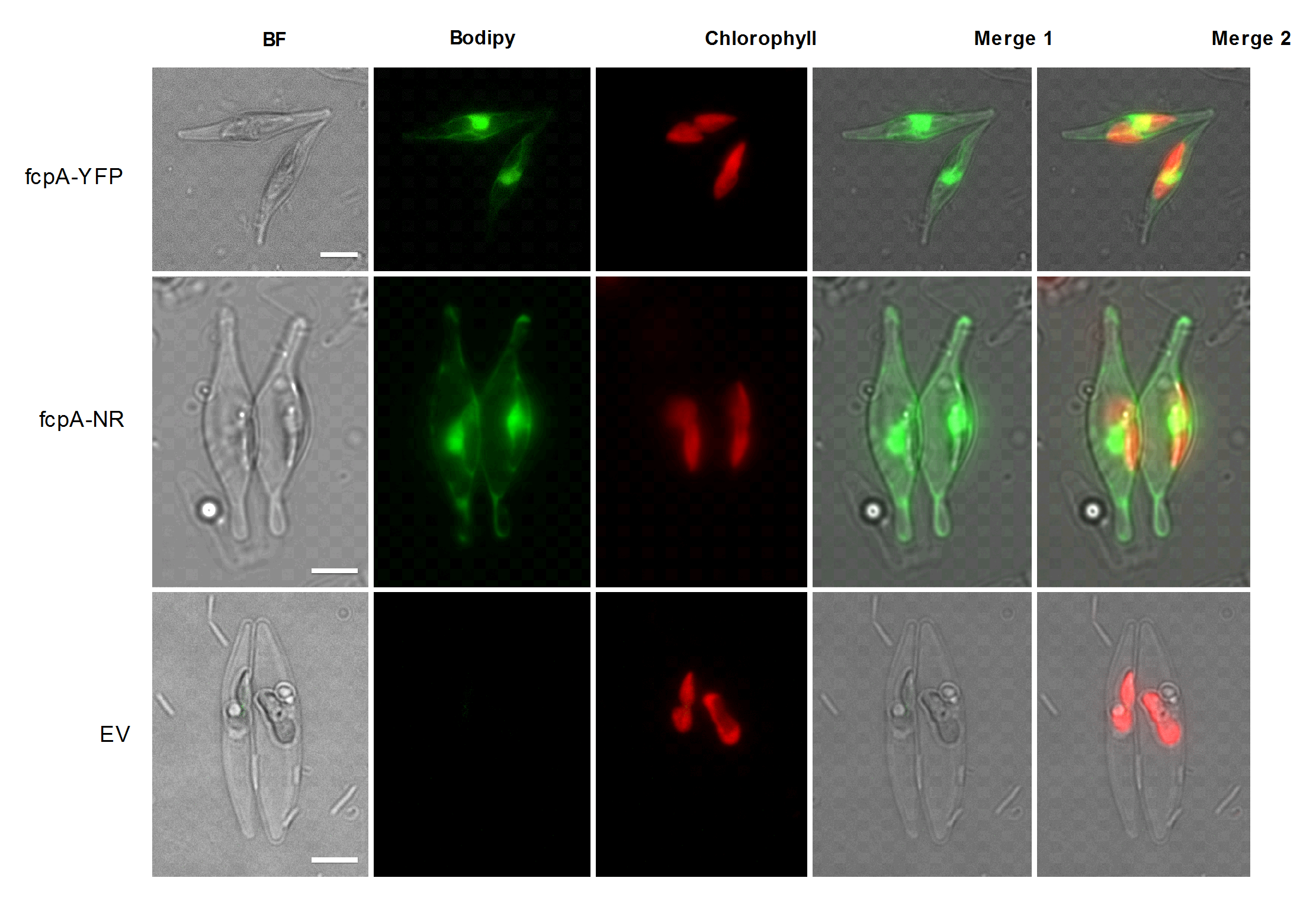

Supplement: Supplementary file 9 — Additional file 9: Figure S9. Comparison of the fcpA and NR promoters in driving YFP expression in Pt4. In both lines, the YFP signal is present in the cytosol. The EV line was used as a negative control and it showed no YFP fluorescence. Images are composites of BF, YFP fluorescence (green), chlorophyll autofluorescence (red) and merged images. Scale bar = 5 µm. BF = bright field, EV = empty vector, NR = nitrate reductase, YFP = yellow fluorescent protein. [file 13068_2017_874_MOESM9_ESM.tif]

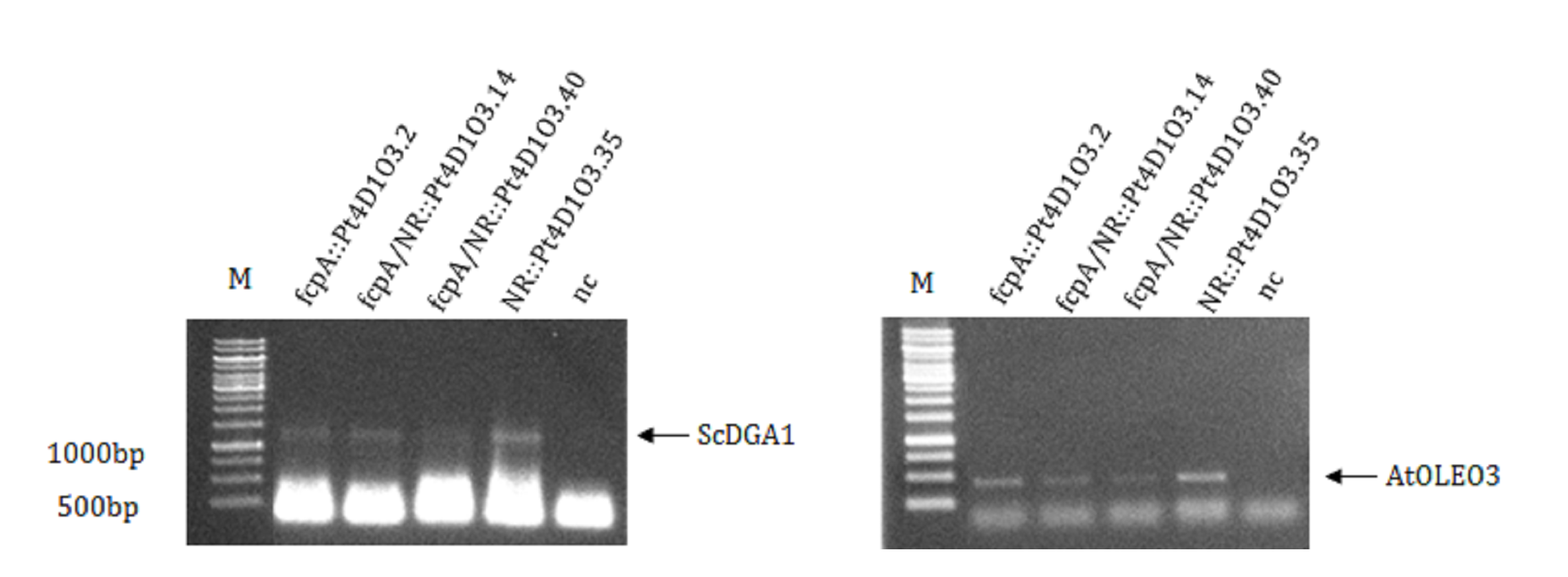

Supplement: Supplementary file 10 — Additional file 10: Figure S10. Semi-quantitative PCR for ScDGA1 and AtOLEO3 co-expressing lines under the fcpA promoter and NR promoter. Screening for lines co-expressing the ScDGA1 and AtOLEO3 genes was by semi-quantitative PCR where the cDNA was used as a template. cDNA extracted from the wild type was used as a negative control. M = marker, nc = negative control, NR = nitrate reductase. [file 13068_2017_874_MOESM10_ESM.tif]
