# Supplementary material for: Heterologous co-expression of a yeast diacylglycerol acyltransferase (ScDGA1) and a plant oleosin (AtOLEO3) as an efficient tool for enhancing triacylglycerol accumulation in the marine diatom Phaeodactylum tricornutum
Source: Biotechnol Biofuels. 2017 Jul 17;10:187. doi: 10.1186/s13068-017-0874-1 (PMC5514505; doi:10.1186/s13068-017-0874-1)
Supplement: Supplementary file 11 — Additional file 11. Nucleotide sequences for ScDGA1 and AtOLEO3 used in this study. [file 13068_2017_874_MOESM11_ESM.docx]

**Additional file 11:** Nucleotide sequences for *ScDGA1* and *AtOLEO3* used in this study.

*ScDGA1* (S000005771) – Codon optimized for *P. tricornutum*

ATGTCGGGCACCTTTAACGATATTCGCCGCCGCAAGAAGGAAGAAGGCTCCCCCACCGCCGGTATTACGGAACGCCACGAAAACAAGTCCTTGTCCTCGATTGACAAGCGTGAACAGACCCTCAAGCCCCAATTGGAATCCTGCTGTCCCCTCGCCACGCCGTTCGAACGTCGTCTTCAGACCCTCGCCGTCGCCTGGCACACGTCCTCGTTTGTCCTCTTCTCCATTTTTACCTTGTTCGCCATCTCGACGCCCGCCTTGTGGGTCCTTGCCATTCCGTACATGATCTACTTCTTTTTCGACCGTTCCCCGGCCACCGGAGAAGTCGTCAACCGCTACTCCCTTCGTTTTCGCTCGCTCCCCATTTGGAAGTGGTACTGCGATTACTTCCCGATTTCCCTTATCAAGACCGTCAACCTCAAGCCCACCTTTACGTTGTCCAAGAACAAGCGTGTCAACGAAAAGAACTACAAGATCCGCTTGTGGCCGACCAAGTACTCCATTAACCTCAAGTCCAACTCGACGATCGACTACCGTAACCAAGAATGCACCGGACCCACGTACTTGTTCGGTTACCACCCGCACGGAATTGGAGCCCTCGGTGCCTTTGGAGCCTTCGCCACCGAAGGTTGTAACTACTCCAAGATCTTTCCCGGCATTCCGATCTCGCTCATGACCTTGGTCACGCAGTTCCACATTCCCCTTTACCGCGATTACCTCTTGGCCCTCGGCATCTCCTCGGTCTCCCGTAAGAACGCCCTTCGCACCCTCTCCAAGAACCAATCGATTTGCATCGTCGTCGGAGGTGCCCGTGAATCCCTTCTCTCCTCGACCAACGGAACGCAGCTCATTTTGAACAAGCGCAAGGGATTCATTAAGTTGGCCATCCAAACCGGTAACATCAACCTCGTCCCCGTCTTTGCCTTCGGTGAAGTCGACTGTTACAACGTCCTCTCCACCAAGAAGGATTCGGTCTTGGGAAAGATGCAGCTTTGGTTTAAGGAAAACTTTGGCTTCACGATTCCCATCTTCTACGCCCGTGGCCTCTTTAACTACGACTTCGGATTGCTTCCCTTTCGTGCCCCGATCAACGTCGTCGTCGGTCGTCCGATTTACGTCGAAAAGAAGATCACCAACCCCCCGGACGATGTCGTCAACCACTTCCACGACTTGTACATTGCCGAATTGAAGCGTCTTTACTACGAAAACCGCGAAAAGTACGGCGTCCCCGATGCCGAACTCAAGATCGTCGGATAA

*AtOLEO3* (AT5G51210) – Codon optimized for *P. tricornutum*

ATGGCCGACCAAACCCGCACGCACCACGAAATGATCTCCCGCGATTCCACCCAAGAAGCCCACCCCAAGGCCCGTCAAATGGTCAAGGCCGCCACCGCCGTCACGGCCGGAGGTTCCCTCTTGGTCCTTTCGGGATTGACCCTTGCCGGTACGGTCATTGCCTTGACCGTCGCCACGCCCCTTCTCGTCATCTTCTCCCCCGTCCTCGTCCCGGCCGTCGTCACCGTCGCCCTCATTATCACGGGTTTCTTGGCCTCCGGCGGATTTGGCATTGCCGCCATCACCGCCTTTTCGTGGCTCTACCGTCACATGACCGGTTCCGGCTCGGACAAGATTGAAAACGCCCGTATGAAGGTCGGATCCCGCGTCCAGGATACCAAGTACGGCCAACACAACATCGGAGTCCAGCACCAGCAAGTCTCGTAA
