# Supplementary material for: Heterologous co-expression of a yeast diacylglycerol acyltransferase (ScDGA1) and a plant oleosin (AtOLEO3) as an efficient tool for enhancing triacylglycerol accumulation in the marine diatom Phaeodactylum tricornutum
Source: Biotechnol Biofuels. 2017 Jul 17;10:187. doi: 10.1186/s13068-017-0874-1 (PMC5514505; doi:10.1186/s13068-017-0874-1)
Supplement: Supplementary file 12 — Additional file 12: Table S1. Restriction site primers used for the cloning of ScDGA1 and AtOLEO3. [file 13068_2017_874_MOESM12_ESM.docx]

| Gene | Primer no. | Primer name | Primer sequence (5' to 3') |
| --- | --- | --- | --- |
| *ScDGA1*(co) | 112 | DGA_SpeI_for | CCGACTAGTATGTCGGGCACCTTTAACG |
|  | 113 | DGA_PacI_rev | CGTTAATTAATTATCCGACGATCTTGAG |
| *AtOLEO3* (co) | 110 | Oleo3_SpeI_for | CGACTAGTATGGCCGACCAAACCCGCACG |
|  | 111 | Oleo3_PacI_rev | CGTTAATTAATTACGAGACTTGCTGGTGC |

**Additional file 12: Table S1.** Restriction site primers used for the cloning of *ScDGA1 a*nd *AtOLEO3*.
