# Supplementary material for: Heterologous co-expression of a yeast diacylglycerol acyltransferase (ScDGA1) and a plant oleosin (AtOLEO3) as an efficient tool for enhancing triacylglycerol accumulation in the marine diatom Phaeodactylum tricornutum
Source: Biotechnol Biofuels. 2017 Jul 17;10:187. doi: 10.1186/s13068-017-0874-1 (PMC5514505; doi:10.1186/s13068-017-0874-1)
Supplement: Supplementary file 13 — Additional file 13: Table S2. Primers used for assessing gene integration and expression of ScDGA1 and AtOLEO3 genes in Pt4. [file 13068_2017_874_MOESM13_ESM.docx]

| Gene | Primer no. | Primer name | Primer sequence (5' to 3') |
| --- | --- | --- | --- |
| *ScDGA1* | 212 | DGA-Codon-for | ATGTCGGGCACCTTTAACGATATTCGC |
|  | 213 | DGA-Codon-rev | TTATCCGACGATCTTGAGTTCGGCATC |
| *AtOLEO3* | 216 | Oleo3co.for | ATGGCCGACCAAACCCGCACG |
|  | 217 | Oleo3co.rev | TTACGAGACTTGCTGGTGCTG |
| Pt Actin | 179 | Pt act1 for | ATGGAGAAGATCTGGCACCACACC |
|  | 180 | Pt act1 rev2 | TCCTTCGAGATCCACATGCTCTGG |

**Additional file 13: Table S2.** Primers used for assessing gene integration and expression of *ScDGA1* and *AtOLEO3* genes in Pt4
